# Repetitive trans-spinal magnetic stimulation improves motor function in rats with spinal cord injury and is associated with upregulation of EphA4 signaling pathway proteins

**DOI:** 10.3389/fneur.2026.1726570

**Published:** 2026-01-16

**Authors:** Hao Liu, Yu Fang, Qian Deng, Jiucai Ye, Jielan Zhou, Rong Luo

**Affiliations:** 1 Department of Pediatrics, West China Second University Hospital, Sichuan University, Chengdu, China; 2 Key Laboratory of Birth Defects and Related Diseases of Women and Children, Ministry of Education, Sichuan University, Sichuan University, Chengdu, China; 3 Sports Medicine Center, West China Hospital, Sichuan University, Chengdu, China; 4 Department of Orthopedics and Orthopedic Research Institute, West China Hospital, Sichuan University, Chengdu, China

**Keywords:** central pattern generator, EphA4, repetitive trans-spinal magnetic stimulation, spinal cord injury, VGLUT2

## Abstract

**Objective:**

Spinal cord injury (SCI) is a highly disabling neurological condition that remains a worldwide challenge in healthcare. Our previous studies found that repetitive trans-spinal magnetic stimulation (rTSMS) applied at the L2 spinal segment yielded the most significant improvement in motor function in rats with SCI; however, the underlying mechanism remains unclear. Recent research indicates that disruption of the EphA4 signaling pathway in glutamatergic interneurons within the spinal cord leads to a loss of motor rhythm and a hopping gait in rats. Conversely, activating the locomotor central pattern generator (CPG) located in the L1-2 spinal segments promotes the recovery of motor function. Thus, by examining the effects of rTSMS on proteins associated with the EphA4 signaling pathway, this study provides novel insights for future investigations into its potential mechanisms of action.

**Methods:**

A multidimensional approach, including behavioral assessments, immunofluorescence, RT-PCR, and Western blotting, was employed to evaluate the effects of rTSMS on motor function in rats with acute SCI. We also assessed its impact on EphA4 mRNA expression levels and the synthesis of related proteins, including VGluT2, EphA4, EphrinB3, and downstream effector molecules Chn1 and Nck1.

**Results:**

The results showed that rTSMS improved the Basso, Beattie, and Bresnahan (BBB) locomotor scores in rats with acute spinal cord injury. It also exerted positive effects on upregulating the expression level of EphA4 mRNA and promoting the synthesis of proteins, including VGluT2, EphA4, EphrinB3, and the downstream effector molecules Chn1 and Nck1.

**Conclusion:**

This study suggests that repetitive trans-spinal magnetic stimulation effectively improves motor function after acute spinal cord injury, concomitant with an upregulation of EphA4 pathway-related proteins, thereby providing a new direction for future mechanistic research.

## 1 Introduction

Spinal cord injury (SCI) is a highly disabling condition resulting from various causes (e.g., traffic accidents, natural disasters) that damage the normal anatomical structure and function of the spinal cord, leading to sensory, motor, and other functional impairments below the level of the lesion (1). According to statistics from the University of Alabama, the annual incidence of SCI in the United States from 2012–2016 was 40–54 cases per million population, with approximately 12,000–17,000 new cases reported each year (2, 3)^.^ As one of the most predominant and prominent functional impairments caused by SCI, motor dysfunction significantly compromises patients’ quality of life.

Currently, therapeutic approaches for spinal cord injury involve a combination of surgical decompression, hormone therapy, neuroprotective agents, cell transplantation, gene therapy, and rehabilitation, which are often applied clinically in combination (1, 4). Repetitive transcranial magnetic stimulation (rTMS), which applies magnetic stimulation to the cerebral cortex, modulates neuronal excitability, induces axonal regeneration and collateral sprouting, promotes neural reorganization, and consequently improves motor function (5–8). In recent years, researchers and clinicians have attempted to apply magnetic stimulation directly to the spinal cord. This approach, known as repetitive trans-spinal magnetic stimulation (rTSMS), has also demonstrated favorable clinical efficacy in improving motor function following spinal cord injury (9, 10).

Our preliminary studies confirmed that rTSMS applied at the L2 segment significantly improved hindlimb motor function in rats (11); however, its mechanism of action remains unclear. Research has revealed that the EphA4 signaling pathway in glutamatergic interneurons of the spinal locomotor central pattern generator (CPG) is critically involved in controlling spinal axon collateral growth and motor rhythm (12). Functioning as a key guidance molecule, EphA4 is expressed on axons of both the corticospinal tract and commissural interneurons. For corticospinal neurons expressing EphA4, their axons sense the repulsive signals from ephrin ligands (such as ephrinB3) distributed in specific concentration gradients within the spinal cord. This interaction precisely guides the axons to their correct target areas in the spinal gray matter, forming an orderly topographic map (13, 14). In EphA4-knockout mice, corticospinal axons exhibit pathfinding errors upon entering the spinal gray matter and fail to establish a precise projection map (15). Fabes et al. (16). reported that following a hemisection spinal cord injury, EphA4 protein extensively accumulated in the proximal stumps of injured corticospinal tract (CST) axons. Concurrently, its ligand, ephrinB2, was markedly upregulated in reactive astrocytes of the glial scar. The interaction of EphA4 and ephrinB2 led to axonal retraction and inhibited regeneration, indicating that EphA4 acts as a key inhibitory factor for CST regeneration following SCI. EphA4 is also expressed by excitatory interneurons (e.g., Vglut2+) in the ventral spinal cord. When the axons of these neurons attempt to project contralaterally, they are strongly repelled by ephrinB3, which is highly concentrated at the spinal midline. This repulsive interaction confines their growth to the ipsilateral side. This mechanism is essential for maintaining the separation of neural circuits controlling the left and right limbs, which underlies the generation of normal alternating gait (12, 17). Loss of this function results in aberrant axonal crossing of the midline, manifesting in animal models as the classic synchronized “hopping gait” (15, 18). Existing studies suggest that EphA4 expression is modulated after spinal cord injury (SCI). Research from Cruz-Orengo et al. (19). indicated that EphA4 mRNA expression was downregulated at days 2 and 4 after spinal cord injury (SCI), upregulated by day 7, and sustained at a certain level by the second week. Similarly, Liu et al. (20). observed a significant downregulation of EphA4 expression in control rats (intrathecal saline injection) at 1, 3, and 7 days post-SCI, followed by an upregulation at 14 and 21 days, also displaying a biphasic pattern. In another study, Chen et al. (21). reported a significant increase in EphA4 expression post-SCI, peaking at 3 days. They further revealed that EphA4, by interacting with its ligand ephrin-B expressed on astrocyte surfaces, regulates the secretion of neurotrophic factors, adhesion molecules, inflammatory cytokines, and glial scar components. Through modulating astrocyte function, EphA4 influences neurite outgrowth and regeneration following SCI. Although the exact timing of EphA4 up- and down-regulation across existing studies is not fully consistent—a variability that may be attributed to factors such as lesion site, severity, and model specifics—the collective evidence indicates an early-phase upregulation of EphA4 expression after SCI to varying degrees. Furthermore, downstream effector molecules of the EphA4 signaling pathway, including *α*-chimerin (Chn1) and the adaptor protein Nck1, play significant roles in axonal growth and guidance. Chn1, a Rac-GTPase-activating protein, mediates EphrinB3/EphA4 forward signaling to regulate corticospinal tract axon guidance and central pattern generator formation. Upon EphA4 receptor activation by EphrinB3, Chn1 inhibits growth cone elongation by inactivating Rac, a positive regulator of neurite outgrowth (22, 23). Nck1 is another crucial downstream effector of EphA4-mediated control of axonal directionality. This adaptor protein is associated with proteins involved in actin cytoskeleton regulation. The Nck1 adaptor couples phosphorylated tyrosine (PTyr) guidance signals to the cytoskeletal rearrangements required for ipsilateral projection of spinal neurons, thereby guiding axonal growth on the ipsilateral side to enable normal limb movement (24).

Notably, in mammals, the spinal central pattern generator (CPG) network controlling hindlimb locomotion is widely accepted to be primarily located within the lumbar enlargement, specifically spanning the L1–L4 segments. Compelling evidence from *in vitro* spinal cord preparations indicates that the L2 segment is a key site for generating the coordinated alternating rhythm of flexion and extension (25). Therefore, this study aimed to employ a multidimensional approach—including behavioral tests, immunofluorescence, RT-PCR, and Western blotting—to evaluate the impact of L2-targeted rTSMS on both motor function and proteins (EphA4, EphrinB3, Chn1, and Nck1) related to the EphA4 signaling pathway in rats with spinal cord injury, thereby providing insights for further mechanistic research.

## 2 Materials and methods

### 2.1 Animals

A total of eighty-six specific pathogen-free (SPF) female Sprague–Dawley rats(3 months old), weighing 260–310 g, were used in the study. The animals were housed at a density of four to five per cage in a quiet, well-ventilated, clean laboratory room. The housing conditions were maintained at a temperature of 22–25 °C and a relative humidity of 40–70%. All the rats, bedding, and feed were purchased from Chengdu Dashuo Biological Technology Co., Ltd. (China).

### 2.2 Establishment of the spinal cord injury model

Surgical instruments were sterilized via high-pressure steam, and the operating room was disinfected with ultraviolet light. The rats were removed from their cages and placed in an induction chamber, where they were exposed to 100% oxygen for 2–3 min. Anesthesia was induced by adjusting a precision vaporizer to deliver 4–5% isoflurane (26). Successful anesthesia was confirmed by the presence of ataxia, pronation, stable and slow respiration, and cessation of limb movement. The rat was then secured in the prone position on the operating table. The tubing was connected from the induction chamber to a nose cone, the vaporizer was adjusted to deliver 1–3% isoflurane, and the flowmeter was set to deliver 1 L/min of oxygen for maintenance anesthesia (27). The dorsal fur was shaved, and the skin was aseptically prepared and draped. One milliliter of saline was administered subcutaneously for hydration. A midline incision approximately 3 cm long was centered on the T10 spinous process. The skin and subcutaneous tissues were incised and dissected layer by layer. The paravertebral muscles were separated and retracted bilaterally. The T9 to T11 laminae were exposed and identified via a combination of ophthalmic scissors and forceps. The T9, T10, and T11 spinous processes were observed as closely spaced clusters. A rongeur was used to perform a complete T10 laminectomy, with partial removal of the T9 and T11 laminae, to fully expose the dorsal spinal dura mater. A triangular needle, held with a needle holder, was passed carefully with its blunt end through the space between the ventral dura and the vertebral body to avoid spinal cord injury or traction. An aneurysm clip was fixed with a clip applicator. The opened clip was advanced through the channel at the T10 level to the contralateral side, ensuring that it spanned the spinal cord completely. The applicator was then released abruptly, resulting in instantaneous, forceful compression of the spinal cord. This was accompanied by spasmodic twitching of the rat’s body and tail. The clip was maintained in place for 10 s before being gently removed. Subsequent observation revealed subdural congestion or hematoma and a clear impression mark on the epidural surface (28). Finally, the wound was irrigated with saline, hemostasis was achieved, and it was sutured in layers to complete the surgery.

### 2.3 Animal grouping and intervention methods

Eighty-six SD rats were randomly divided into four groups: the sham-operated group (SO, *n* = 11), the control group (CON, *n* = 25), the repetitive trans-spinal magnetic stimulation group (rTSMS, *n* = 25), and the sham stimulation group (S-rTSMS, *n* = 25). The rats in the SO group underwent T10 laminectomy with complete exposure of the dura mater but did not receive spinal cord clip compression.

Beginning on the fourth postoperative day, the rats in the SO and rTSMS groups received rTSMS treatment at the L2 spinal segment via a Magstim magnetic stimulator (Magstim Company Ltd., UK). The center of the stimulation coil was positioned close to the L2 spinal segment. The rats in the S-rTSMS group received sham stimulation, wherein the coil was rotated 90 degrees relative to the spinal cord while maintaining identical stimulation parameters to those of the rTSMS group. This configuration ensured that no magnetic field passed through the rat’s spinal cord, whereas the coil’s vacuum cooling system generated auditory cues identical to those of active stimulation. Treatments were administered daily at 3:00 p.m. The stimulation parameters were as follows: frequency of 5 Hz, intensity at 75% of the maximum output (1.5 T), with each train lasting 5 s followed by a 2-s inter-train interval. Each session consisted of 10 trains, which were administered once daily, 5 days per week, for six consecutive weeks.

### 2.4 Quantification of Basso, Beattie, and Bresnahan (BBB) scores

Hindlimb motor function was assessed via the Basso, Beattie, and Bresnahan (BBB) open-field locomotor rating scale, where a score of 0 indicates complete paralysis and a score of 21 represents normal function (29). Behavioral evaluations were performed by two investigators blinded to the treatment groups at designated time points: one day before surgery and at 2, 4, and 6 weeks after surgery.

### 2.5 Collection of spinal cord tissue samples

At 2, 4, and 6 weeks post-surgery, five rats were randomly selected from the CON, rTSMS, and S-rTSMS groups, and two rats were selected from the SO group. The selected rats were anesthetized via intraperitoneal injection of 3% sodium pentobarbital (100 mg/kg). Following successful anesthesia, the dorsal skin was incised as described in the modeling procedure. The laminae and spinous processes surrounding the L2 segment were carefully removed to fully expose the spinal cord. The bilateral spinal nerve roots were dissected via a glass dissecting needle, and a segment of approximately 1 cm in length centered on the L2 segment was excised. The tissue sample was immediately placed into a cryotube, labeled, and submerged in liquid nitrogen. After collection, all samples were rapidly transferred to a − 80 °C freezer for subsequent storage and analysis.

At the 6-week post-operative time point, three rats were randomly selected from each group for transcardial perfusion to collect spinal cord tissue. A segment approximately 1 cm in length, centered on the L2 spinal segment, was harvested. Following anesthesia via the method described previously, the rat was secured in a prone position on the operating platform. An abdominal incision was made along the costal margin. The xiphoid process was retracted upward, and the thoracic cavity was opened by making a cut along both sides of the sternum. The pericardium was carefully removed to fully expose the heart and the ascending aorta, allowing visualization of the left ventricle. The perfusion needle was inserted into the apex of the left ventricle, and rapid perfusion with 150 mL of normal saline was initiated. Upon distension of the ascending aorta, the needle tip was advanced into the aortic arch and secured. An outlet was then created by incising the right atrial appendage. The perfusate was switched to a universal, neutral-buffered tissue fixative (4% paraformaldehyde in PBS), following a protocol of initial rapid perfusion followed by a slower rate. Perfusion was terminated when the liver turned pale, and strong limb convulsions accompanied by body twisting were observed. The dorsal skin was subsequently incised along the vertebral column according to the modeling procedure. The laminae and spinous processes surrounding the L2 segment were removed to fully expose the spinal cord. The bilateral spinal nerve roots were carefully dissected and transected. A segment of spinal cord tissue approximately 1 cm in length, centered on the L2 level, was carefully excised. The harvested tissue sample was placed in 4% paraformaldehyde fixative for 24 h at 4 °C for subsequent analysis.

### 2.6 Immunofluorescence

Fixed spinal cord tissues were dehydrated through a graded sucrose series (20 and 30%), embedded, and frozen. Serial sections were cut at a thickness of 30 μm using a cryostat. The sections were then incubated in a blocking solution containing 5% bovine serum albumin (BSA) and 0.1% Triton X-100 in 0.1 M Tris-buffered saline (TBS) for 60 min. This was followed by incubation with primary antibodies for 12 h at 4 °C. The sections were subsequently incubated with appropriate secondary antibodies for 2 h at room temperature and finally counterstained with DAPI (1:500) for 15 min at room temperature. After three washes with PBS, the sections were mounted with anti-fade mounting medium. For immunofluorescence, the following primary antibodies were used: a rabbit polyclonal anti-EphA4 antibody (Product # 21875-1-AP, Proteintech; 1:50 dilution), which targets a protein of approximately 120 kDa, and a rabbit monoclonal anti-VGluT2 antibody (Product # DF13296, Affinity Biosciences; 1:200 dilution), targeting a protein of about 64 kDa. All sections were imaged using a Zeiss Axioscan 7 slide scanner. The exposure times were set at 7 ms for DAPI, 15 ms for EGFP (corresponding to the VGluT2 signal), and 250 ms for Cy3 (corresponding to the EphA4 signal). The numbers of EphA4-positive neurons, VGluT2-positive neurons, and EphA4/VGluT2 double-positive neurons were quantified via ImageJ software, and their respective ratios were calculated. The antibody dilution ratios used were as follows: EphA4 (1:50), VGluT2 (1:200), Cy3-conjugated secondary antibody (1:200), and EGFP-conjugated secondary antibody (1:200).

### 2.7 RT–PCR

The relative expression level of EphA4 mRNA was detected via quantitative real-time RT–PCR (qRT–PCR), which was performed according to the procedure described by Ogawa et al. (30). Briefly, spinal cord tissues were pulverized in liquid nitrogen and homogenized in TRIzol reagent for 30 s, and total RNA was extracted following the manufacturer’s instructions. The extracted RNA was dissolved in a suitable volume of diethylpyrocarbonate (DEPC)-treated distilled water. The RNA concentration and integrity were assessed by measuring the A260/A280 ratio via a spectrophotometer and denaturing agarose gel electrophoresis. One microgram of total RNA was reverse-transcribed into cDNA via oligo-dT primers. Subsequently, real-time PCR amplification was performed using the synthesized cDNA. The 20 μL reaction mixture contained 2 μL of buffer, 2 μL of cDNA, 1.5 μL of dNTPs, 1 μL each of forward and reverse primers, 1 μL of fluorescence probe, and 1 μL of hot-start enzyme. The reactions were run on an ABIPRISM 7000 Sequence Detection System (Applied Biosystems, USA). After initial predenaturation at 95 °C for 2 min, 40 amplification cycles were conducted as follows: denaturation at 94 °C for 30 s, annealing at 58 °C (for GAPDH) or 55 °C (for EphA4) for 30 s, and extension at 72 °C for 1 min. The PCR amplification employed previously published primer sequences (31):

EphA4:

Forward: 5’-CAGAGGTAAGGGTAGGAGGC-3’.

Reverse: 5’-AGCAGTGTAGCGAGCACAAC-3’.

GAPDH (internal control):

Forward: 5’-AACTTTGGCATTGTGGAAGG-3’.

Reverse: 5’-GGATGCAGGGATGATGTTCT-3’.

The relative expression level of EphA4 mRNA was determined using the comparative 2^(–ΔΔCt) method. The GAPDH gene served as the endogenous reference for normalization, and the control (CON) group was designated as the calibrator.

The calculation procedure was as follows: the ΔCt value for each sample was calculated as ΔCt = Ct < sub > EphA4</sub > − Ct < sub > GAPDH</sub>. The ΔΔCt for each experimental sample (from the rTSMS, S-rTSMS, or SO groups) was then derived by subtracting the average ΔCt of the CON calibrator group: ΔΔCt = ΔCt < sub > sample</sub > − ΔCt < sub > CON (mean)</sub>. Finally, the fold change in gene expression was calculated as 2^(–ΔΔCt).

### 2.8 Western blot analysis

At 2, 4, and 6 weeks after SCI, a 1 cm segment of spinal cord tissue centered on the L2 level was harvested and placed in a grinding tube. One milliliter of protein extraction reagent (RIPA buffer containing protease inhibitors, RIPA: PMSF = 100:1) was added, and the tissue was homogenized until a uniform lysate was obtained. For protein extraction, the homogenate was centrifuged, and the supernatant was collected. The supernatant was mixed with loading buffer and denatured by heating at 100 °C for 5 min. The protein concentration was determined via the bicinchoninic acid (BCA) assay. Protein samples from each group were separated via 10% SDS–polyacrylamide gel electrophoresis (SDS–PAGE) and subsequently transferred onto polyvinylidene fluoride (PVDF) membranes. The membranes were sequentially incubated with specific primary antibodies followed by the corresponding secondary antibodies. The protein bands were visualized via an enhanced chemiluminescence (ECL) substrate, and the band intensity was quantified via densitometric analysis via ImageJ software. The optical density of immunoreactive bands corresponding to EphA4, EphrinB3, Chn1, Nck1, and the loading control *β*-actin was determined by densitometric analysis using ImageJ software. For each sample, the relative expression level of a protein of interest was calculated by normalizing the densitometry value of its band to that of the β-actin band from the same lane.

### 2.9 Statistical analysis

All experimental data were statistically analyzed via SPSS software (version 19.0). The normality of continuous variables was assessed via the Kolmogorov–Smirnov test. Normally distributed data are presented as the means ± standard deviations. The behavioral data were compared via repeated-measures analysis of variance (ANOVA). For other data, one-way ANOVA was used for comparisons among multiple group means. If the assumption of homogeneity of variance was met, *post hoc* pairwise comparisons were performed via the least significant difference (LSD) test. In cases of unequal variances, Tamhane’s T2 test was applied. A value of *p* ≤ 0.05 was considered statistically significant.

## 3 Results

### 3.1 rTSMS treatment improves motor function in rats with acute spinal cord injury

**Figure 1:**
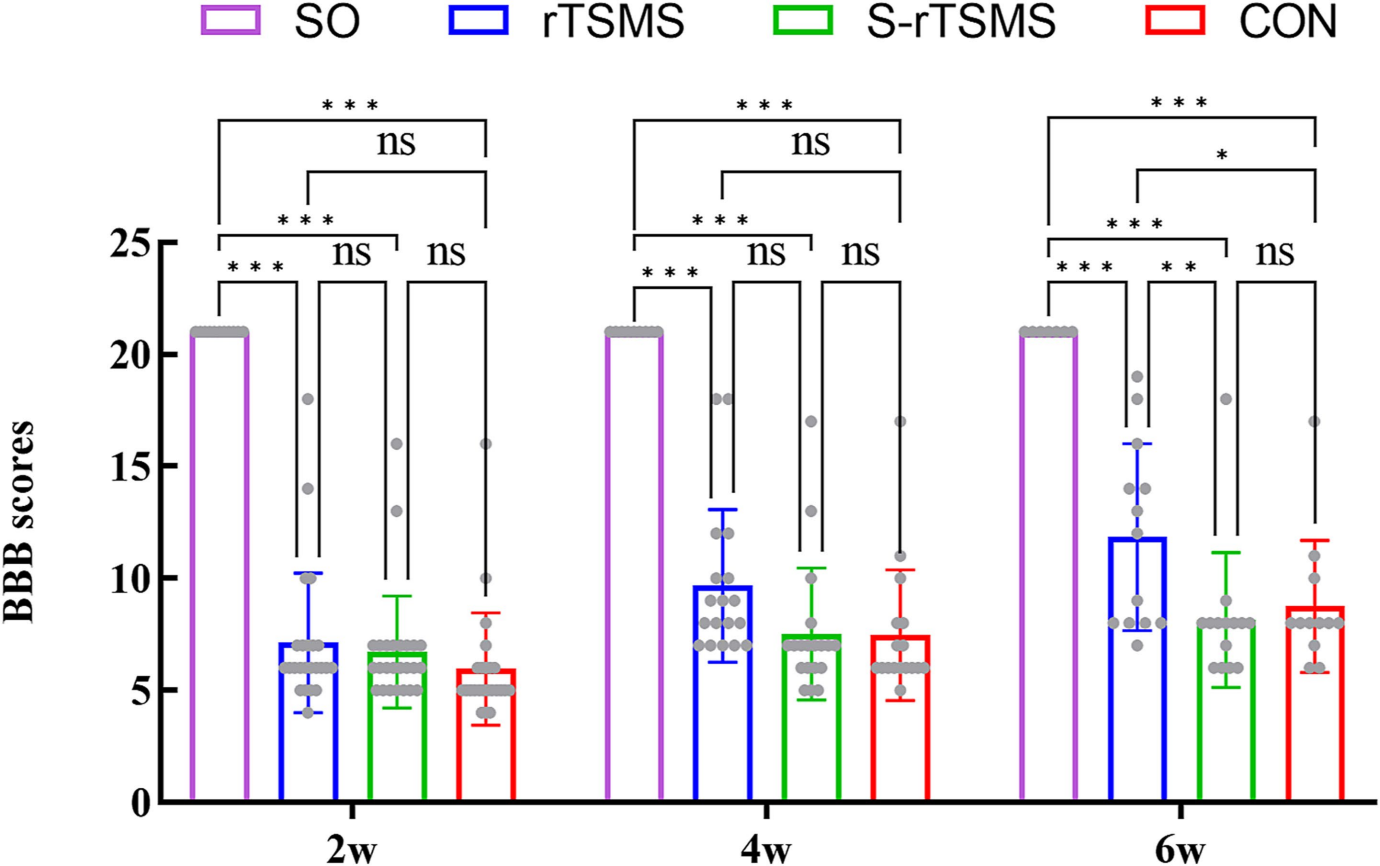
BBB scores. RTSMS treatment for 6 weeks significantly improved motor function in the rats. The data are presented as the means ± standard deviations. *p < 0.05, **p < 0.01, ***p < 0.001; ns, not significant. CON, ontrol group; rTSMS, repetitive trans-spinal magnetic stimulation group; S-rTSMS, sham stimulation group; SO, sham-operated group.

The Basso, Beattie, and Bresnahan (BBB) locomotor rating scale was used to assess motor function in the rats. All the rats achieved a BBB score of 21 on the day before surgery. Following SCI, the BBB scores in the rTSMS, S-rTSMS, and CON groups decreased rapidly, with rats exhibiting typical paraplegic symptoms and hindlimb motor dysfunction during crawling. No statistically significant differences in BBB scores were observed among the rTSMS, S-rTSMS, and CON groups at 2 and 4 weeks post-injury. However, at the 6-week time point, the BBB score in the rTSMS group was significantly greater than that in both the CON and S-rTSMS groups (*p* < 0.05). This result indicates that 6 weeks of rTSMS treatment significantly improved motor function recovery. In contrast, no significant difference was found between the CON and S-rTSMS groups (Figure 1), suggesting that the auditory cues generated by the coil cooling system during sham stimulation did not produce a pseudoeffect on motor function.

### 3.2 rTSMS treatment enhances EphA4 mRNA expression in rats with acute SCI

**Figure 2:**
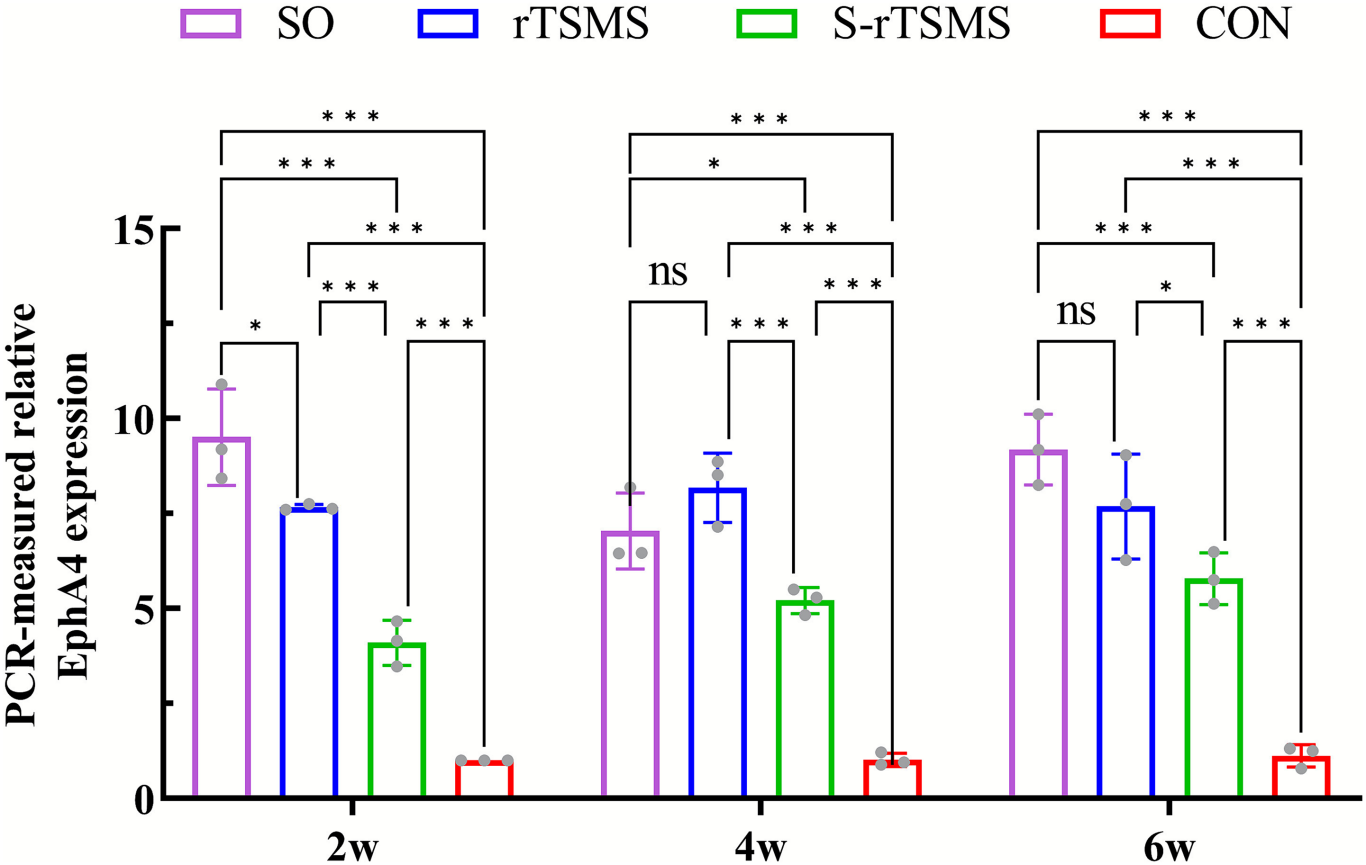
Relative expression level of EphA4 mRNA detected by RT–PCR. rTSMS treatment effectively increased EphA4 mRNA expression in rats with acute SCI, with this effect potentially peaking at approximately week 4. The data are presented as the means ± standard deviations. *p < 0.05, **p < 0.01, ***p < 0.001; ns, not significant. CON, control group; rTSMS, repetitive trans-spinal magnetic stimulation group; S-rTSMS, sham stimulation group; SO, sham-operated.

The relative expression level of EphA4 mRNA was detected by RT–PCR at 2, 4, and 6 weeks post-injury. The relative expression of EphA4 mRNA in both the rTSMS and S-rTSMS groups was significantly greater than that in the CON group at all time points (*p* < 0.001). Notably, the expression level in the S-rTSMS group was also significantly elevated compared with that in the CON group (p < 0.001), a result that differed from the behavioral findings. Furthermore, the relative expression of EphA4 mRNA in the rTSMS group was significantly greater than that in the S-rTSMS group at all time points (*p* < 0.05). By weeks 4 and 6, no statistically significant difference was observed between the rTSMS and SO groups (Figure 2). These results suggest that the rTSMS effectively enhances EphA4 mRNA expression in SCI rats, and this effect may peak at approximately 4 weeks post-treatment.

### 3.3 rTSMS treatment promotes the synthesis of EphA4, EphrinB3, and the downstream proteins Chn1 and Nck1 in the L2 spinal segment of rats with acute SCI

**Figure 3:**
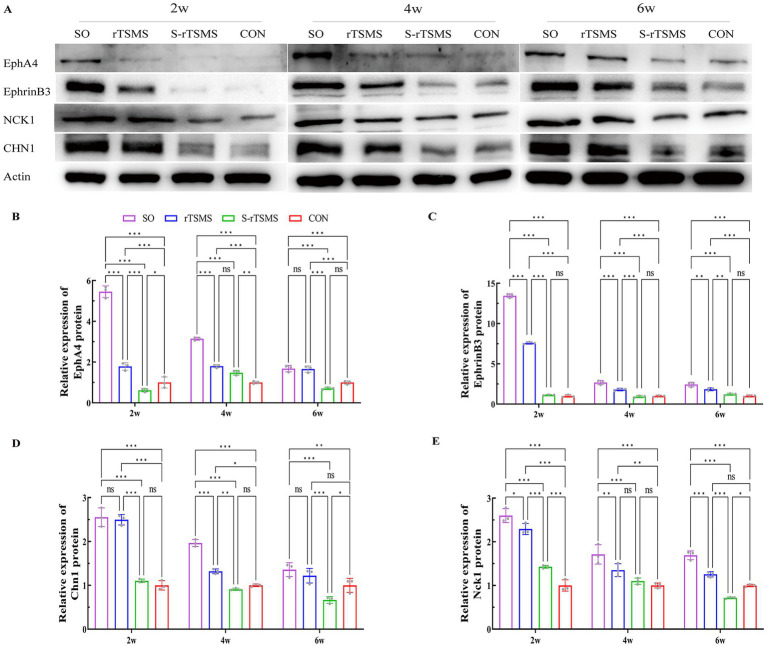
Protein levels of EphA4, EphrinB3, and the downstream proteins Chn1 and Nck1. rTSMS treatment effectively promoted the synthesis of EphA4, EphrinB3, Chn1, and Nck1 in rats with acute SCI. **(A)** Representative western blot images of EphA4, EphrinB3, Chn1, and Nck1 at weeks 2, 4, and 6. **(B)** Relative expression level of EphA4 at each time point (*n* = 3). **(C)** Relative expression level of EphrinB3 at each time point (*n* = 3). **(D)** Relative expression level of Chn1 at each time point (*n* = 3). **(E)** Relative expression level of Nck1 at each time point (*n* = 3). The data are presented as the means ± standard deviations. *p < 0.05, **p < 0.01, ***p < 0.001; ns, not significant. CON, control group; rTSMS, repetitive transspinal magnetic stimulation group; S-rTSMS, sham stimulation group; SO, sham-operated group.

The protein levels of EphA4, EphrinB3, and the downstream effectors Chn1 and Nck1 were detected via Western blotting at 2, 4, and 6 weeks post-injury (Figure 3A). EphA4 is a key protein that guides ipsilateral axonal growth in the spinal cord. At week 2, the expression of EphA4 was significantly greater in both the rTSMS and S-rTSMS groups than in the CON group (*p* < 0.05), with the rTSMS group showing significantly greater expression than the S-rTSMS group (*p* < 0.001). At week 4, EphA4 expression in the rTSMS group remained significantly greater than that in the CON group (p < 0.001), but no significant difference was observed compared with that in the S-rTSMS group. By week 6, EphA4 expression in the rTSMS group was significantly greater than that in both the CON and S-rTSMS groups (p < 0.001), whereas no significant difference was detected between the S-rTSMS and CON groups (Figure 3B). These results suggest that EphA4 protein expression increases at 2 weeks post-SCI but tends to decrease thereafter; rTSMS treatment appeared to attenuate this decrease over time. EphrinB3 is a critical protein that prevents axons from crossing the midline barrier of the spinal cord (13). At all three time points, the expression of EphrinB3 in the rTSMS group was significantly greater than that in both the CON and S-rTSMS groups (*p* < 0.01). No significant difference was observed between the S-rTSMS and CON groups (Figure 3C). These findings indicate that rTSMS effectively promotes the synthesis of EphrinB3. Chn1 is an important protein that inhibits axonal growth cone elongation in motor circuits (32). At weeks 2 and 4, Chn1 expression was significantly greater in the rTSMS group than in both the CON and S-rTSMS groups (*p* < 0.05), with no significant difference between the S-rTSMS and CON groups. At week 6, Chn1 expression in the S-rTSMS group was significantly lower than that in both the CON and rTSMS groups (p < 0.05), while no significant difference was found between the rTSMS and CON groups (Figure 3D). These results suggest that rTSMS also promotes the synthesis of the downstream protein Chn1, at least transiently. Nck1 is a crucial downstream effector that mediates EphA4-controlled axonal guidance. At week 2, Nck1 expression was significantly increased in both the rTSMS and S-rTSMS groups compared with the CON group (*p* < 0.001), with the rTSMS group showing significantly higher expression than the S-rTSMS group (*p* < 0.001). At week 4, Nck1 expression in the rTSMS group remained significantly higher than that in the CON group (*p* < 0.01), but no significant differences were detected between the S-rTSMS group and either the CON or the rTSMS group. By week 6, Nck1 expression in the S-rTSMS group was significantly lower than that in both the CON and rTSMS groups (*p* < 0.05), while no significant difference was found between the rTSMS and CON groups. The temporal pattern of Nck1 expression resembled that of EphA4, with an initial increase followed by a decrease, which was also attenuated by rTSMS treatment (Figure 3E).

**Figure 4:**
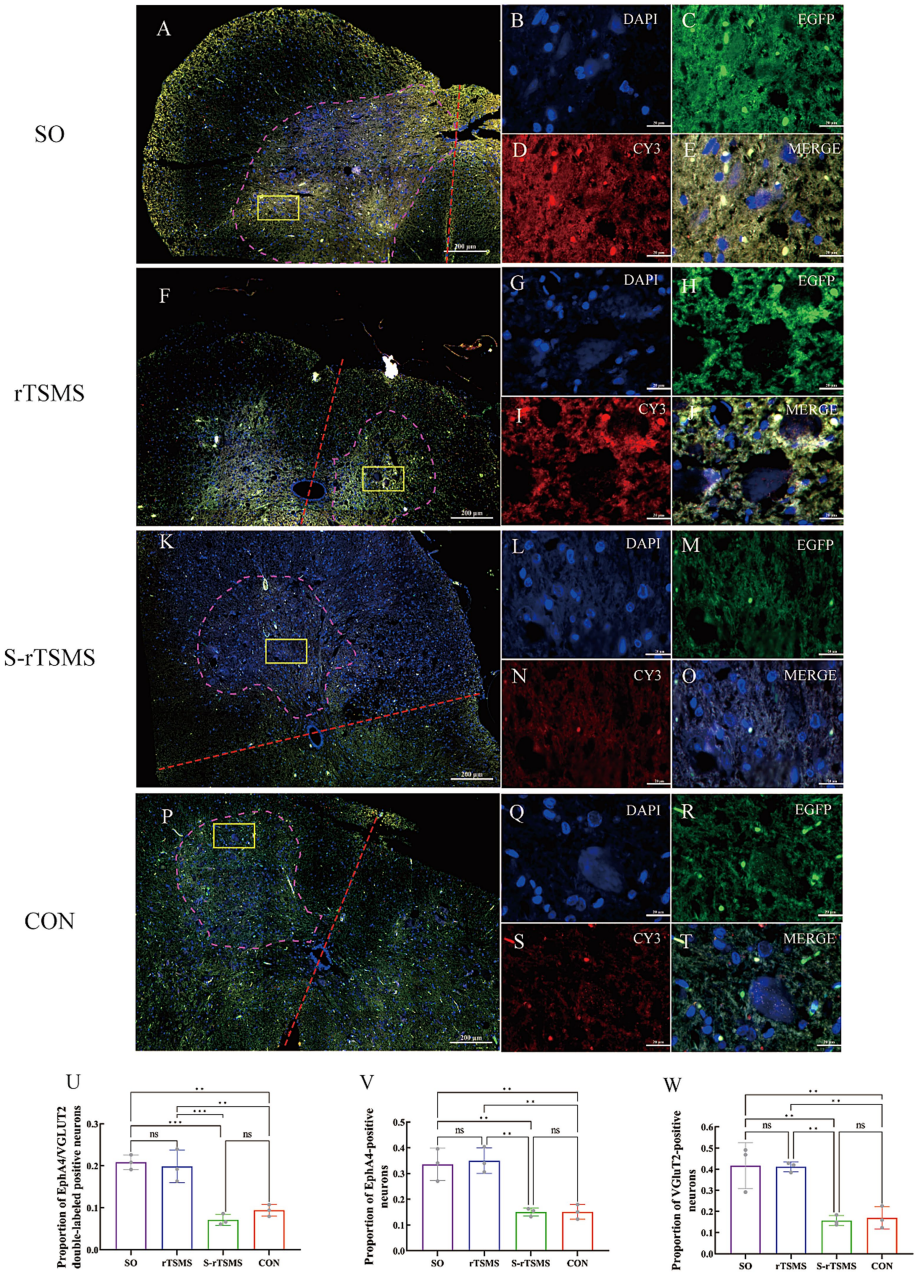
Coexpression of EphA4 and VGluT2 detected by immunofluorescence. Six weeks of rTSMS treatment effectively promoted the synthesis of EphA4 and VGluT2 in cells of the ventral horn of the spinal cord in rats with acute SCI. Panels **(A,F,K,P)** show low-magnification immunofluorescence overviews of the spinal cord. The ventral horn cell region is outlined by a purple dashed line. A red dashed line indicates the dorsoventral axis. The yellow rectangle marks the specific area selected for high-magnification imaging in the subsequent panels. The scale bar represents 200 μm. High-magnification views (Panels **B–E**,**G–J**,**L–O**,**Q–T**) are displayed at a uniform scale of 20 μm (scale bars shown). In these panels, immunopositive signals for EphA4 (detected with Cy3) appear in red, while those for VGluT2 (detected with EGFP) appear in green. Co-localization of EphA4 and VGluT2 immunoreactivity results in a yellowish signal. **(U)** Proportion of EphA4/VGluT2 double-labeled immunopositive neurons relative to the total cell count (*n* = 3). **(V)** Proportion of EphA4-labeled neurons relative to the total cell count (*n* = 3). **(W)** Proportion of VGluT2-labeled neurons relative to the total cell count (*n* = 3). The data are presented as the means ± standard deviations. *p < 0.05, **p < 0.01, ***p < 0.001; ns, not significant. CON, control group; rTSMS, repetitive transspinal magnetic stimulation group; S-rTSMS, sham stimulation group; SO, sham-operated group.

The coexpression of EphA4 and VGluT2 was detected via immunofluorescence labeling. In the low-magnification immunofluorescence overviews (Figures 4A,F,K,P), the yellow boxes indicate the regions selected for high-magnification imaging. The nuclei are stained blue with DAPI (Figures 4B,G,L,Q). EphA4 immunofluorescence labeling revealed that Cy3-labeled EphA4 immunopositive material, which emits red fluorescence, appeared as large red aggregates densely localized within the cytoplasm, with no expression in the nucleus. It was primarily distributed in the neuronal cells of the spinal cord ventral horn laminae VIII and IX (Figures 4D,I,N,S). VGluT2 immunofluorescence labeling revealed that EGFP-labeled VGluT2 immunopositive material, which emits green fluorescence, appeared as fine green granules densely concentrated in the cytoplasm and absent from the nucleus. It was mainly distributed in neuronal cells and nerve terminals within the spinal cord ventral horn laminae VIII, IX, and X, as well as the dorsal horn laminae I and II (Figures 4C,H,M,R). EphA4/VGluT2 double immunofluorescence labeling was performed. Due to the colocalization of EphA4- and VGluT2-immunopositive materials, the overlapping green and red fluorescence resulted in yellowish fluorescence. This was predominantly observed in neuronal cells, including motor neurons and interneurons, in the ventral horn of the spinal cord (Figures 4F,J,O,T). at week 6, the proportions of EphA4/VGluT2 double-labeled immunopositive neurons, EphA4-labeled neurons, and VGluT2-labeled neurons in the ventral horn of the rTSMS group were significantly greater than those in the CON and S-rTSMS groups (*p* < 0.01) and were not significantly different from those in the SO group (Figures 4U–W).

## 4 Discussion

### 4.1 Repetitive transcranial magnetic stimulation effectively ameliorates motor dysfunction resulting from spinal cord injury

Repetitive transcranial magnetic stimulation (rTMS) modulates neuronal excitability, induces axonal regeneration and collateral sprouting (5), activates cortical areas and circuit reorganization, promotes the reconstruction of damaged spinal circuits, and improves motor function (9). Currently, rTMS is widely used in clinical practice and has demonstrated efficacy in improving motor function after SCI (33)^.^ In contrast, repetitive trans-spinal magnetic stimulation (rTSMS) applies magnetic stimulation directly to the spinal cord and has been increasingly explored in recent years for patients with SCI. For example, Brito et al. (10) applied an rTSMS to a 23-year-old patient with SCI caused by neuromyelitis optica. This patient, who presented with complete paralysis and sensory loss in both lower limbs, showed improvements in walking independence, balance, and mobility after 12 rTSMS sessions. Furthermore, numerous studies using animal models of SCI have reported that rTSMS improves motor function (34–38) and alleviates SCI-related neuropathic pain (39). Consistent with these findings, our study also demonstrated significant recovery of motor function in rats with acute SCI following 6 weeks of rTSMS treatment.

### 4.2 Repetitive trans-spinal magnetic stimulation may promote the expression of proteins associated with the EphA4 signaling pathway

While the beneficial role of noninvasive rTSMS in improving motor function after SCI has been reported, its underlying mechanisms remain incompletely elucidated. rTSMS is known to improve motor function in SCI animals through multiple other mechanisms. Zhai et al. (34) reported that rTSMS may promote motor recovery in SCI rats by enhancing microglial clearance of myelin debris—a process potentially mediated by low-density lipoprotein receptor-related protein-1—thereby suppressing neuroinflammation and glial scar formation. Furthermore, a study by Robac et al. (36) demonstrated that the rTSMS can also modulate lesion characteristics by reducing cystic cavity formation and improving axonal survival post-SCI, leading to improved motor function. rTSMS has also been shown to activate peripheral nerve axons, reducing spasticity and enhancing motor function in paralyzed patients (35). Additionally, research by Chalfouh et al. (37) revealed that, in addition to modulating lesion scarring, promoting axonal regeneration, and supporting neuronal survival, rTSMS promoted the proliferation and differentiation of spinal cord stem cells.

Both our preliminary research and the current study revealed that magnetic stimulation applied directly to the L2 spinal segment effectively improved motor function in rats with SCI. Furthermore, the present study offers preliminary molecular-level observations on the effects of rTSMS on proteins associated with the EphA4 signaling pathway. These findings may provide a valuable reference for further mechanistic investigation. The spinal locomotor central pattern generator (CPG) is a circuit within the spinal cord capable of generating rhythmic motor output. It essentially consists of a group of excitatory and inhibitory interneurons that form a specialized neural network structure. Studies have shown that this circuit is closely associated with functions such as jumping, walking, and breathing (40). As a manifestation of spinal cord plasticity, the CPG can be activated by interventions such as body weight-supported treadmill training, functional electrical stimulation, and epidural electrical stimulation (41–45). Magnetic stimulation, a noninvasive neuromodulation technique, can regulate neural networks by modulating network excitability, activating neural feedback loops, and facilitating activity-dependent synaptic plasticity (9, 46). Therefore, it is plausible that magnetic stimulation may similarly possess the capacity to activate the spinal locomotor CPG. In mammals, the spinal central pattern generator (CPG) network controlling hindlimb locomotion is widely accepted to be primarily located within the lumbar enlargement, specifically spanning the L1–L4 segments. Compelling evidence from *in vitro* spinal cord preparations indicates that the L2 segment is a key site for generating the coordinated alternating rhythm of flexion and extension (25). We hypothesize that magnetic stimulation applied to the L2 spinal segment may promote motor function recovery by activating the CPG, thereby inducing plasticity-related changes in neurons.

Accumulating evidence suggests that glutamatergic interneurons, which serve as core excitatory interneurons within the CPG network, play a dominant role in the initiation and regulation of locomotor activity (47, 48). Among these, vesicular glutamate transporters 1 and 2 (VGluT1 and VGluT2) are highly specific markers for glutamatergic neurons and are located at the terminals of glutamatergic axons. VGluT2 is primarily distributed in subcortical structures, including the thalamus and brainstem (49). We therefore hypothesize that VGluT2 may play a critical role in the activation process of the CPG circuit. Our immunofluorescence results support this finding, showing a significantly greater proportion of VGluT2-positive material in ventral horn neuronal cells in the rTSMS group than in the CON and S-rTSMS groups, indicating that magnetic stimulation may promote the synthesis of VGluT2. This finding is consistent with the study by Leydeker et al. (38), which reported that rTSMS increases the release of the neurotransmitter glutamate, thereby increasing the excitability and plasticity of spinal neural circuits.

Furthermore, studies have revealed that the axon guidance molecule EphA4 and its ligand EphrinB3 play significant roles within the mammalian spinal locomotor CPG network (17). Importantly, the EphA4 signaling pathway in excitatory glutamatergic neurons is essential for proper wiring of the motor network. Selective disruption of this pathway leads to a hopping gait, likely caused by abnormal synchronous rhythms associated with increased specific midline crossing of glutamatergic axon terminals in the ventral spinal cord (12). Based on the above rationale, we conducted an exploratory study to observe the effects of rTSMS applied at the L2 spinal segment on motor function and EphA4 pathway-related proteins in rats.

Our immunofluorescence results suggested that the proportions of EphA4-labeled neurons and EphA4/VGluT2 double-positive neurons in the ventromedial region of the spinal cord in the rTSMS group were comparable to those in the SO group and significantly greater than those in the CON and S-rTSMS groups. Furthermore, investigations via RT–PCR and Western blotting revealed that the expression level of EphA4 mRNA was significantly greater in the rTSMS group than in the CON and S-rTSMS groups at weeks 2 and 4. By week 6, it remained significantly elevated compared with that of the CON group, but no significant difference was observed compared with that of the S-rTSMS group. The expression level of EphA4 protein was not significantly different from that in the S-rTSMS group at week 2; however, overall, it was greater in the rTSMS group than in both the S-rTSMS and CON groups across all three time points, a trend generally consistent with the mRNA data. This pattern suggests that magnetic stimulation may more potently enhance the expression of this gene within the first 4 weeks post-SCI, with the effect gradually attenuating thereafter. Notably, the expression level of EphA4 mRNA in the S-rTSMS group was greater than that in the CON group at 2, 4, and 6 weeks but significantly lower than that in the rTSMS group. Similarly, at week 4, EphA4 protein expression in the S-rTSMS group was greater than that in the CON group but significantly lower than that in the rTSMS group. The observed effects in the sham group are hypothesized to be due to incomplete magnetic isolation. To prevent such interference and improve methodological rigor, we plan to adopt control measures in future work, such as auditory-only sham stimulation or the use of a Faraday cage, to fully isolate the sham group from any active magnetic field.

Additionally, the protein expression of EphrinB3, which serves as the midline barrier, was significantly greater in the rTSMS group than in both the CON and S-rTSMS groups at all three time points, peaking at week 2 before it gradually decreased. These findings suggest that magnetic stimulation may exert effects on both EphA4 and EphrinB3. While the influences on both proteins appear to be synchronized within the first 2 weeks, the effect on EphA4 seems to be more sustained. Therefore, we propose that magnetic stimulation applied at the L2 spinal segment may effectively promote EphA4 gene expression and protein synthesis within the first 4 weeks following SCI. Concurrently, magnetic stimulation may upregulate the synthesis of EphrinB3 protein primarily within the initial 2 weeks, after which this effect gradually diminishes.

Furthermore, we examined key downstream effector molecules of EphA4 in cortical and spinal motor circuits, including *α*-chimerin (Chn1) and Nck1. Our results revealed that the levels of both Chn1 and Nck1 in the rTSMS group were greater than those in the S-rTSMS group across all three time points, a pattern similar to the synthesis of the EphA4 protein. These data suggest that magnetic stimulation may upregulate the expression of the downstream effector molecules, Chn1 and Nck1. Collectively, stimulation at the L2 spinal segment appears to promote the upregulation of proteins associated with the EphA4 pathway, offering novel insights for future mechanistic investigations.

## 5 Conclusion

Our study suggests that repetitive trans-spinal magnetic stimulation (rTSMS), as a non-invasive neuromodulation technique, effectively improves motor function in rats with acute spinal cord injury and promotes the expression of proteins associated with the EphA4 signaling pathway, thereby offering valuable insights for further mechanistic research. Although some experiments have yielded positive results, several limitations should be addressed in future work. First, while we observed an upregulation of proteins associated with the EphA4 signaling pathway, we have not directly confirmed whether this change is accompanied by a concurrent increase in the excitability of the CPG circuit or at the spinal segment L2. Therefore, in the next phase, we will employ cre reporter lines to investigate changes specifically within glutamatergic interneurons. Concurrently, we will utilize the Nicolet EDX system to record motor-evoked potentials (MEPs), somatosensory-evoked potentials (SEPs), and the HMax/MMax ratio. Furthermore, metabolic activity in the CPG at the L2 segment will be assessed using 18F-FDG-Micro-PET/CT imaging. By comparing the effects of rTSMS on cortical versus L2 spinal segment excitability and tracking changes in L2 CPG metabolism, this integrated approach will allow us to specifically explore the impact of rTSMS on spinal CPG excitability. Second, to ensure the specificity of the sham stimulation and rule out confounding effects from any residual magnetic field, future experiments will employ refined control procedures. This will be achieved either by delivering only the recorded acoustic cues without generating a magnetic field or by conducting sham stimulation within a magnetically shielded enclosure. Additionally, to address the limited statistical power associated with the current sample size in molecular assays, we will significantly increase the number of biological replicates in subsequent experiments.

## Data Availability

The datasets presented in this study can be found in online repositories. The names of the repository/repositories and accession number(s) can be found in the article/Supplementary material.
