# Supplementary figures and images for: Repetitive trans-spinal magnetic stimulation improves motor function in rats with spinal cord injury and is associated with upregulation of EphA4 signaling pathway proteins
Source: Front Neurol. 2026 Jan 16;17:1726570. doi: 10.3389/fneur.2026.1726570 (PMC12855122; doi:10.3389/fneur.2026.1726570)

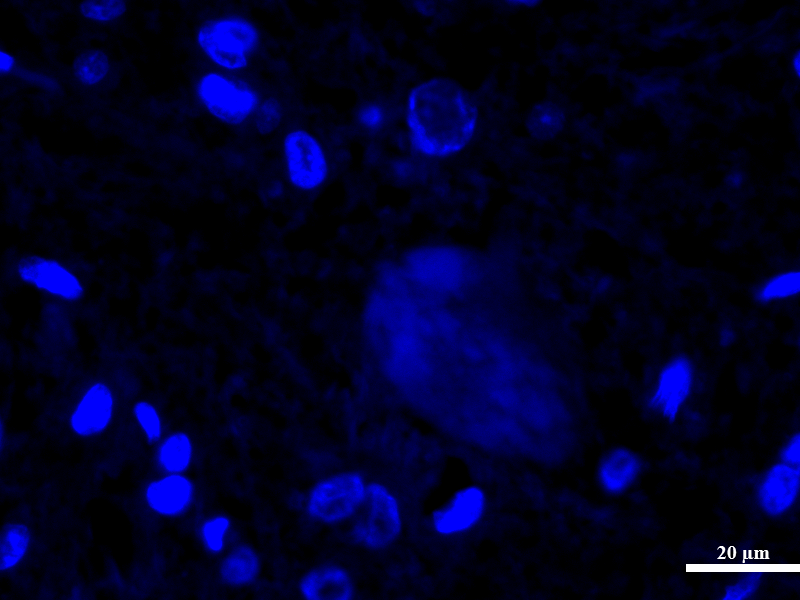

Supplement: Supplementary file 1 [file Data_Sheet_1.ZIP › Datasets/Immunofluorescence images/CONTROL/CON1.tif]

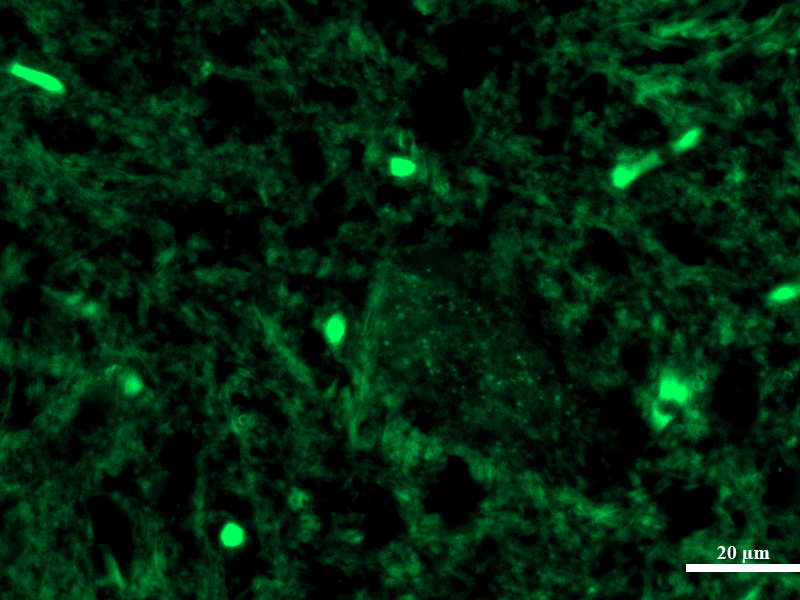

Supplement: Supplementary file 1 [file Data_Sheet_1.ZIP › Datasets/Immunofluorescence images/CONTROL/CON2.tif]

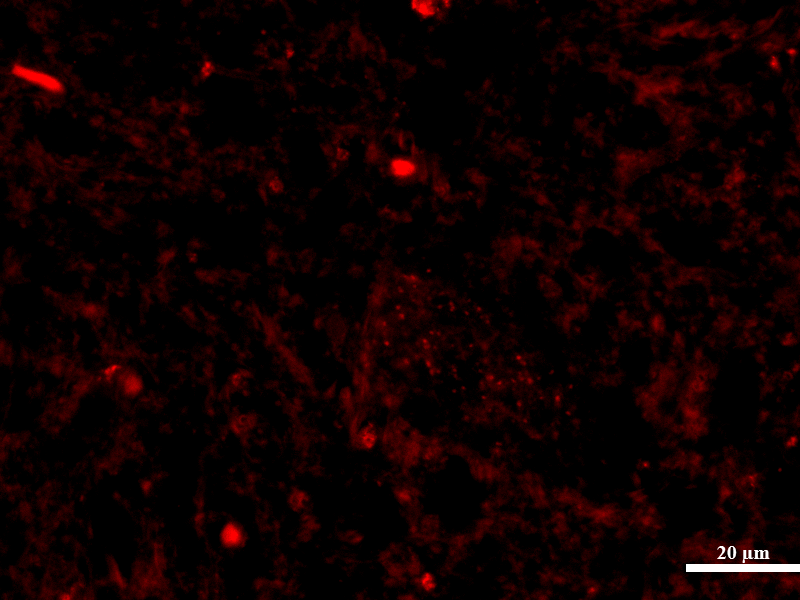

Supplement: Supplementary file 1 [file Data_Sheet_1.ZIP › Datasets/Immunofluorescence images/CONTROL/CON3.tif]

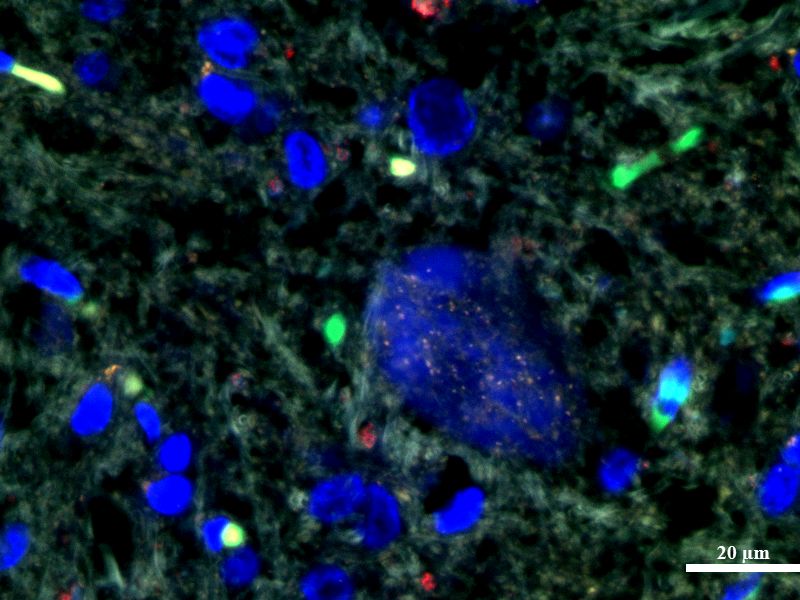

Supplement: Supplementary file 1 [file Data_Sheet_1.ZIP › Datasets/Immunofluorescence images/CONTROL/CON4.tif]

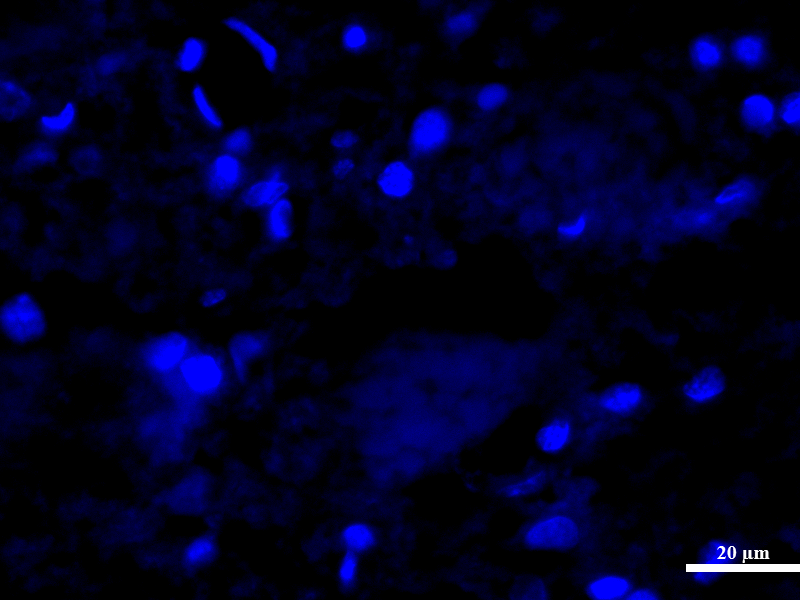

Supplement: Supplementary file 1 [file Data_Sheet_1.ZIP › Datasets/Immunofluorescence images/rTSMS/rTSMS1.tif]

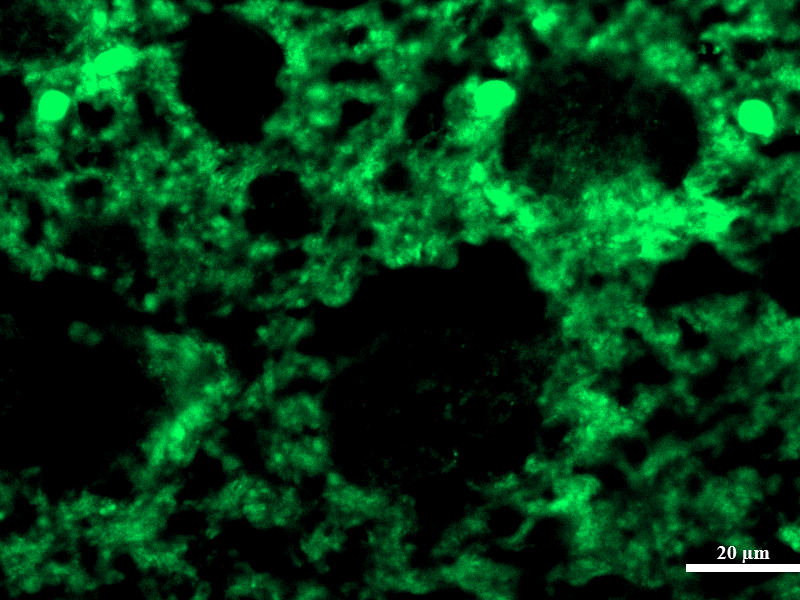

Supplement: Supplementary file 1 [file Data_Sheet_1.ZIP › Datasets/Immunofluorescence images/rTSMS/rTSMS2.tif]

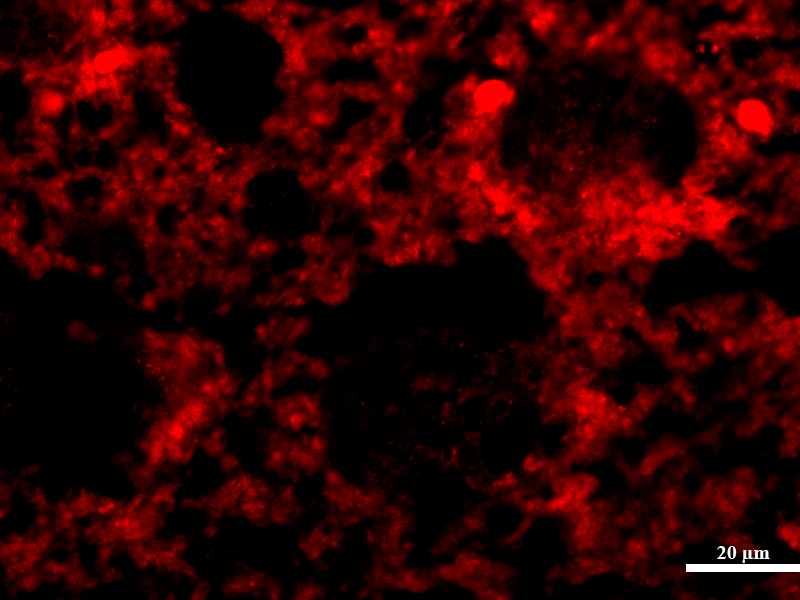

Supplement: Supplementary file 1 [file Data_Sheet_1.ZIP › Datasets/Immunofluorescence images/rTSMS/rTSMS3.tif]

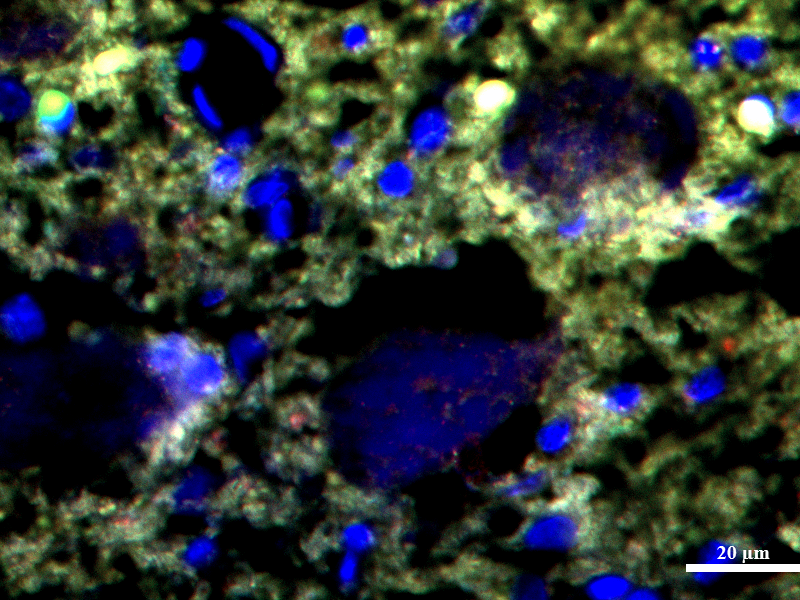

Supplement: Supplementary file 1 [file Data_Sheet_1.ZIP › Datasets/Immunofluorescence images/rTSMS/rTSMS4.tif]

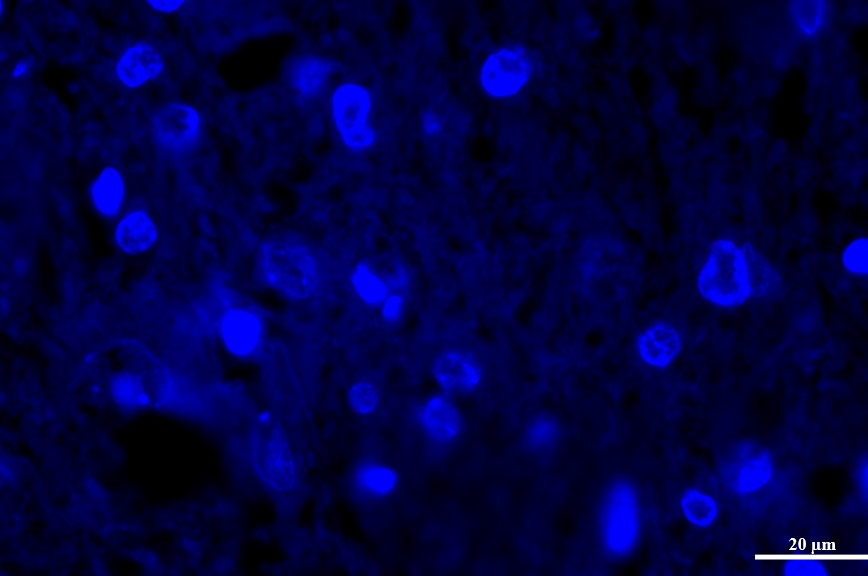

Supplement: Supplementary file 1 [file Data_Sheet_1.ZIP › Datasets/Immunofluorescence images/S-rTSMS/S-rTSMS1.jpg]

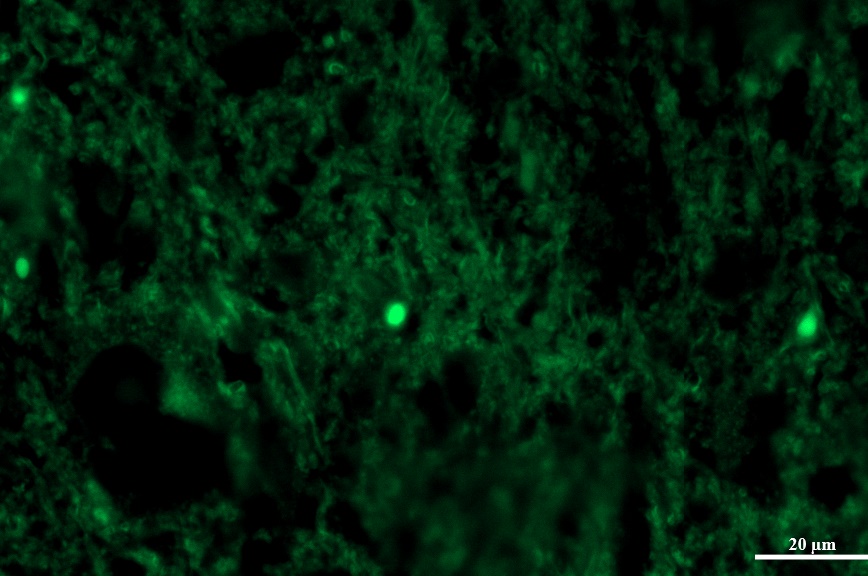

Supplement: Supplementary file 1 [file Data_Sheet_1.ZIP › Datasets/Immunofluorescence images/S-rTSMS/S-rTSMS2.jpg]

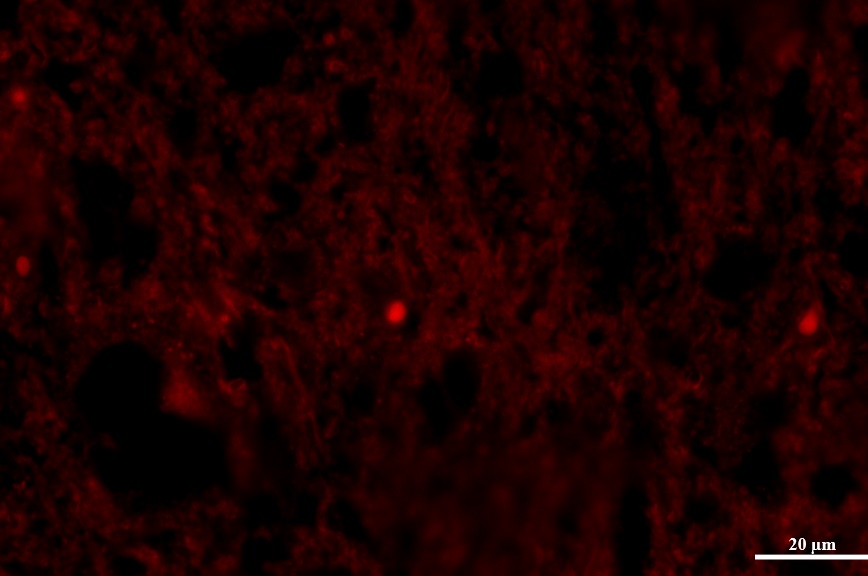

Supplement: Supplementary file 1 [file Data_Sheet_1.ZIP › Datasets/Immunofluorescence images/S-rTSMS/S-rTSMS3.jpg]

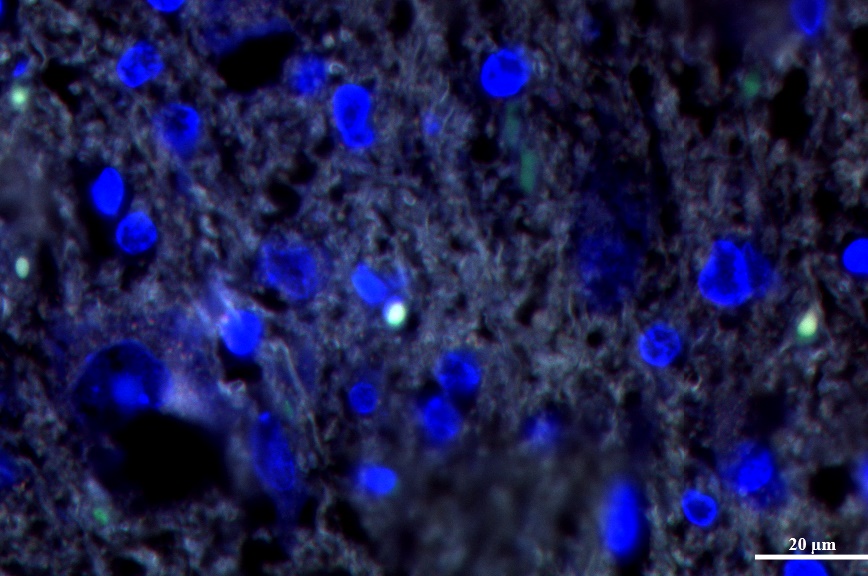

Supplement: Supplementary file 1 [file Data_Sheet_1.ZIP › Datasets/Immunofluorescence images/S-rTSMS/S-rTSMS4.jpg]

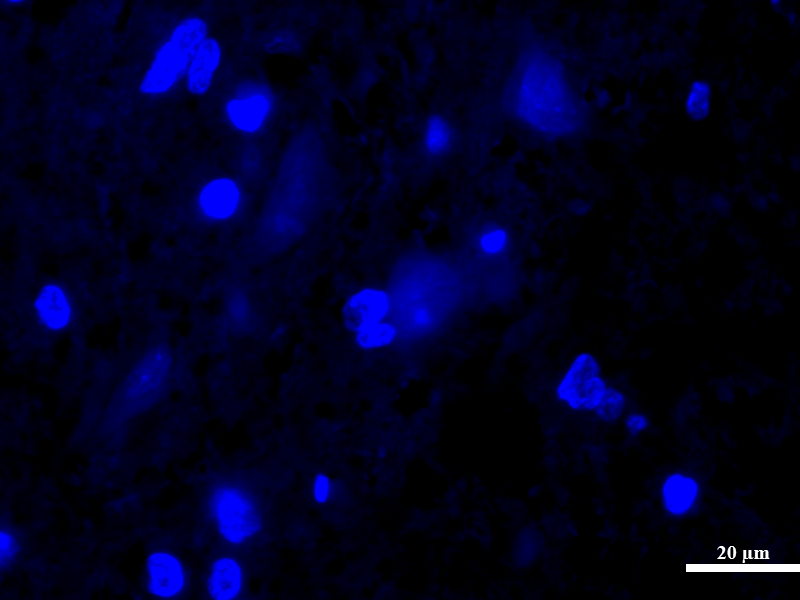

Supplement: Supplementary file 1 [file Data_Sheet_1.ZIP › Datasets/Immunofluorescence images/SO/SO1.tif]

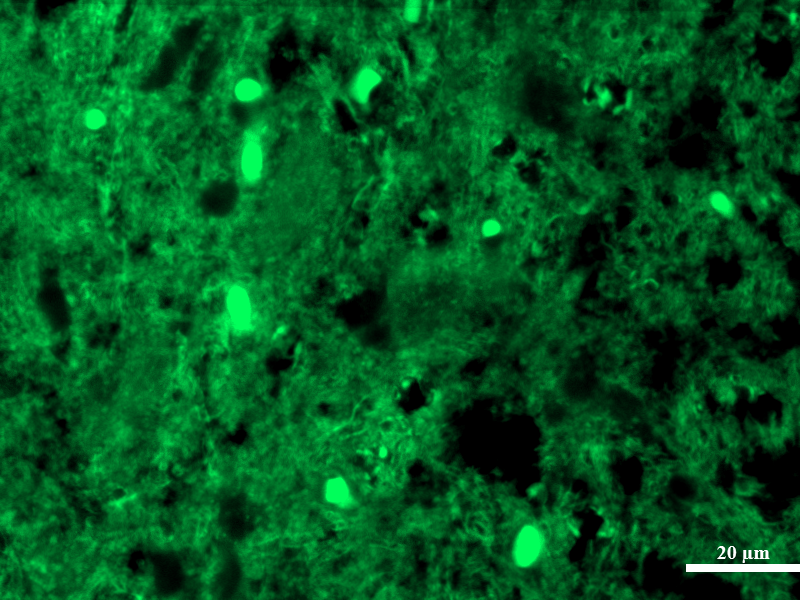

Supplement: Supplementary file 1 [file Data_Sheet_1.ZIP › Datasets/Immunofluorescence images/SO/SO2.tif]

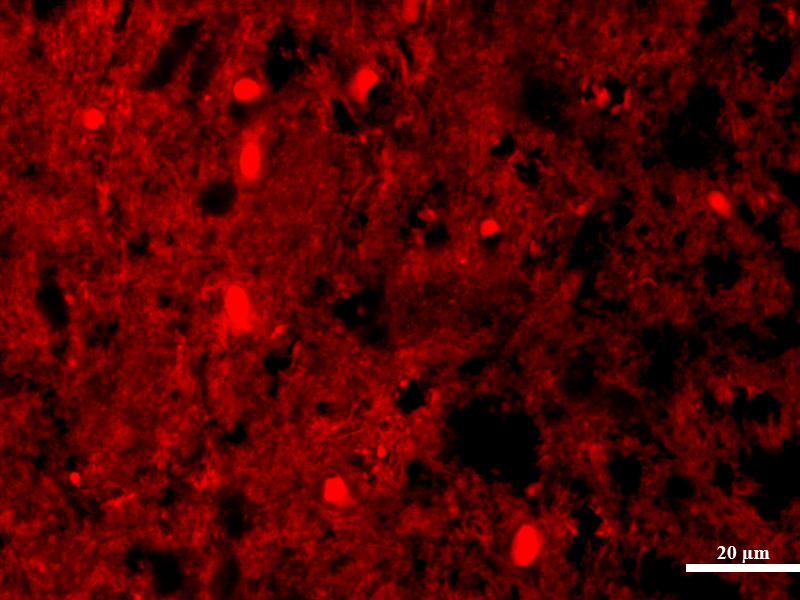

Supplement: Supplementary file 1 [file Data_Sheet_1.ZIP › Datasets/Immunofluorescence images/SO/SO3.tif]

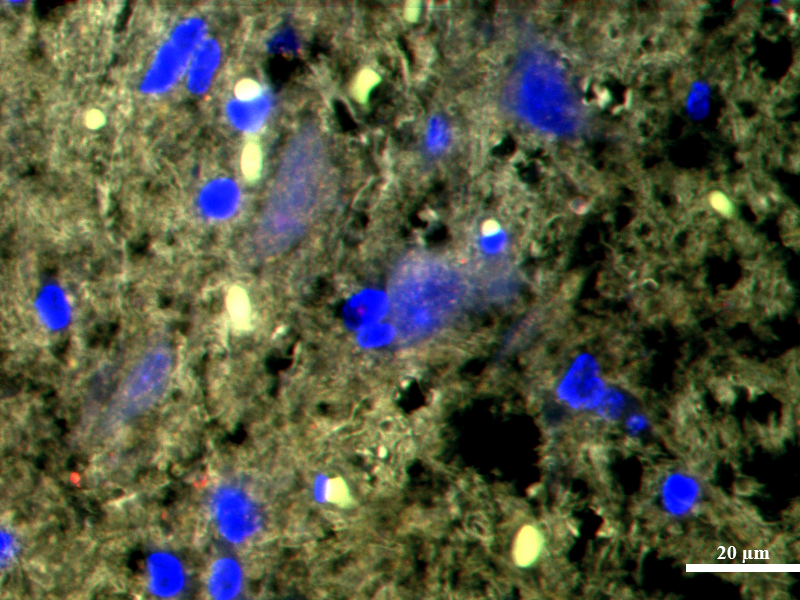

Supplement: Supplementary file 1 [file Data_Sheet_1.ZIP › Datasets/Immunofluorescence images/SO/SO4.tif]

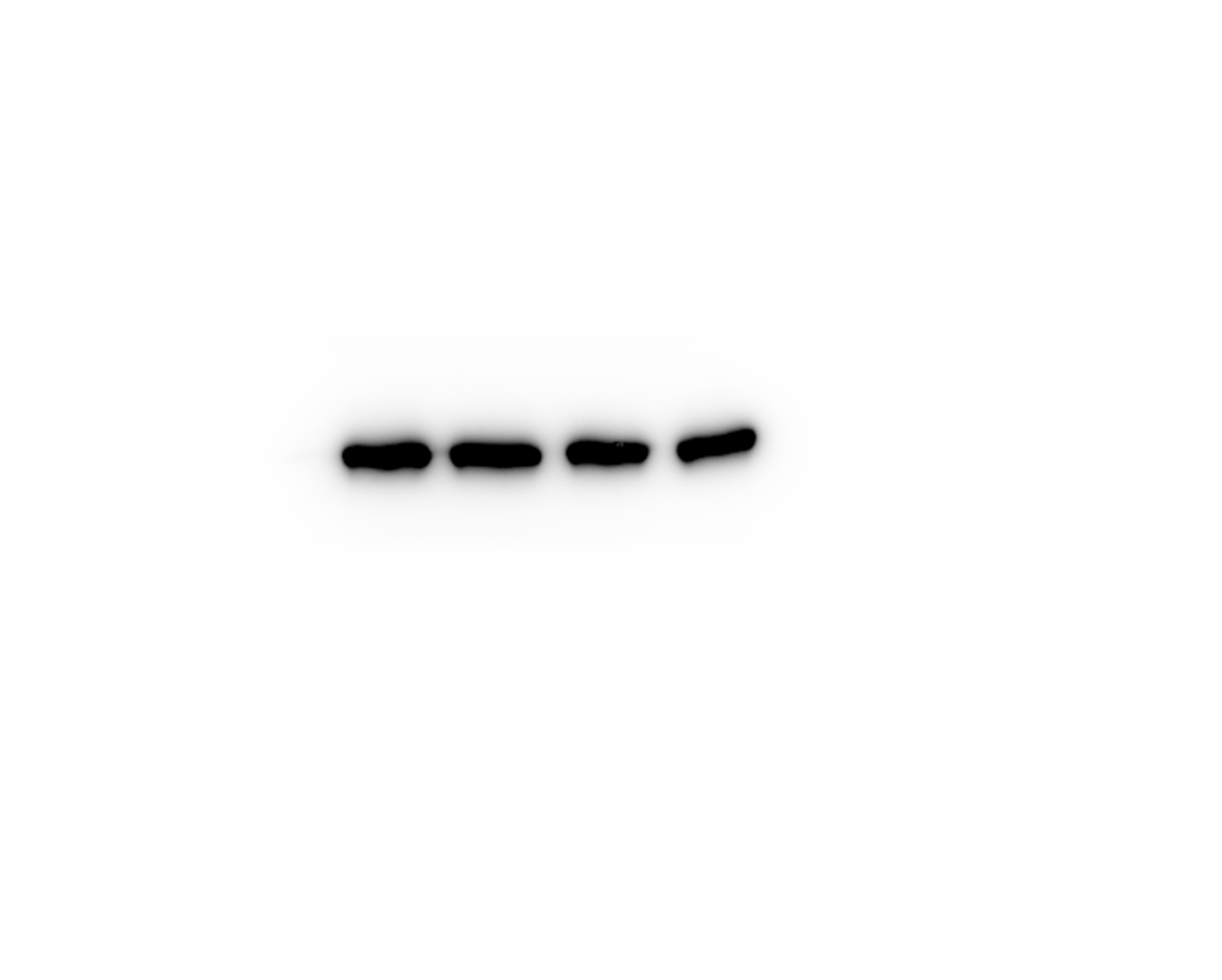

Supplement: Supplementary file 1 [file Data_Sheet_1.ZIP › Datasets/Western blot images/Actin/actin(4W).Tif]

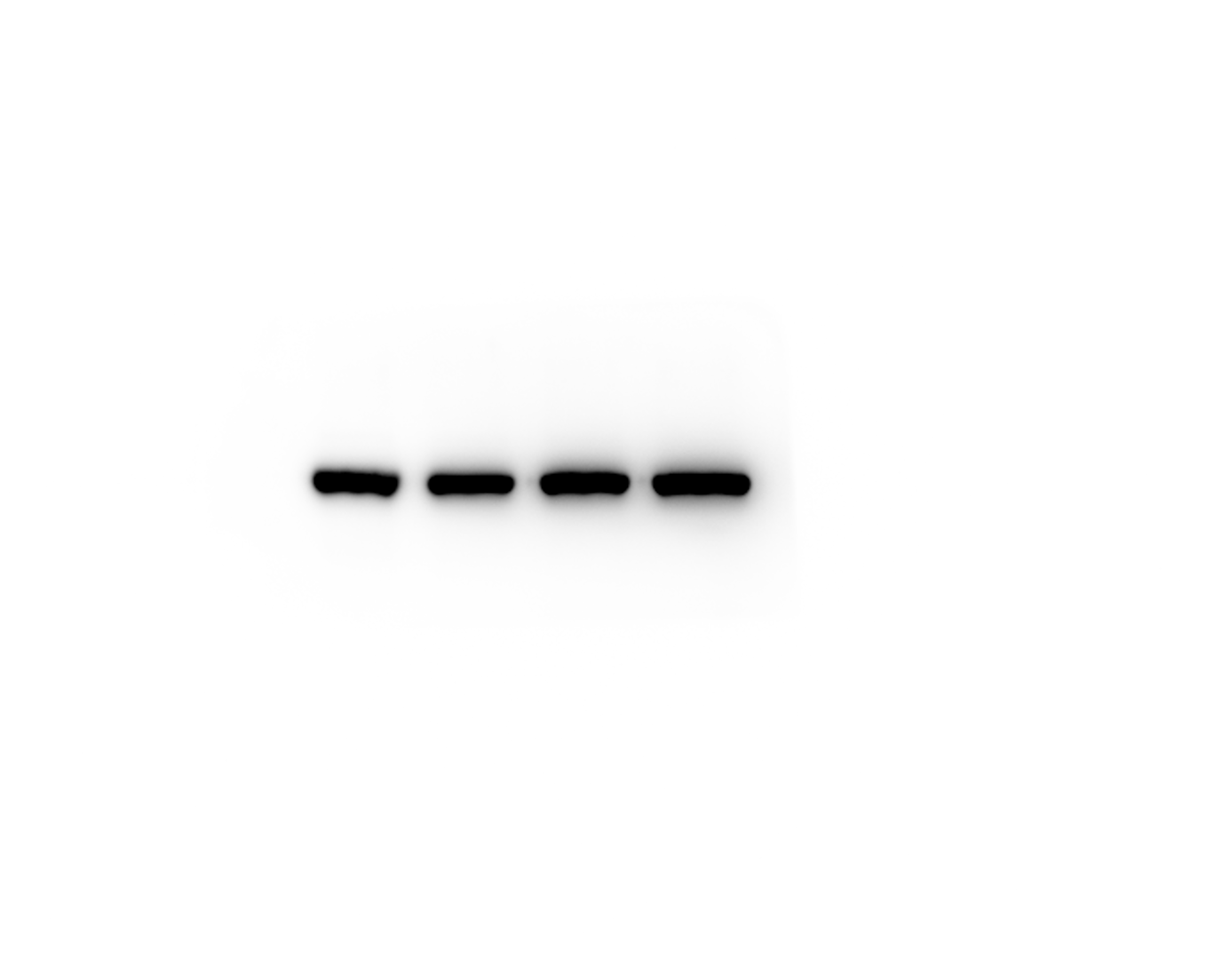

Supplement: Supplementary file 1 [file Data_Sheet_1.ZIP › Datasets/Western blot images/Actin/actin1(2W).Tif]

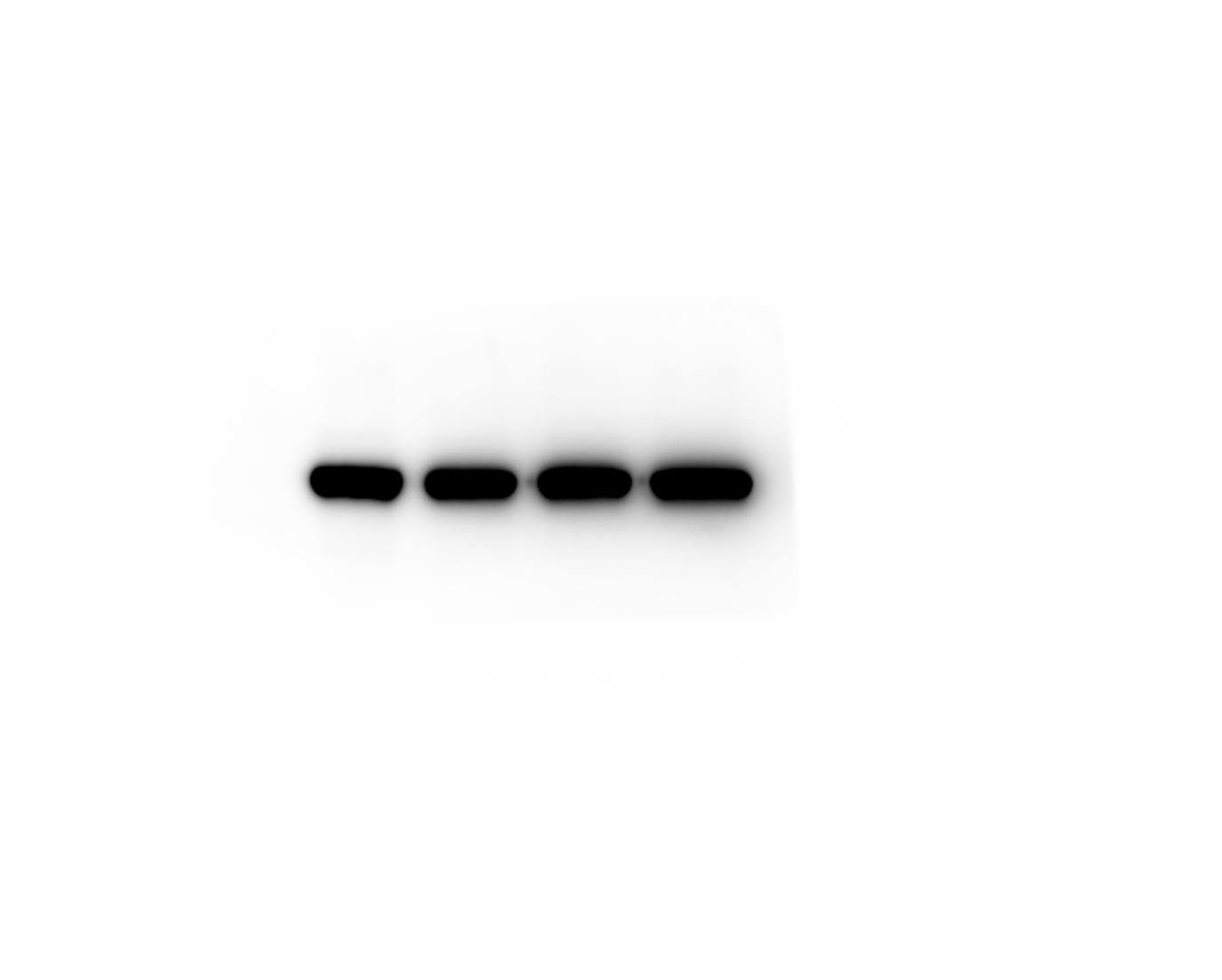

Supplement: Supplementary file 1 [file Data_Sheet_1.ZIP › Datasets/Western blot images/Actin/actin1(6W).Tif]

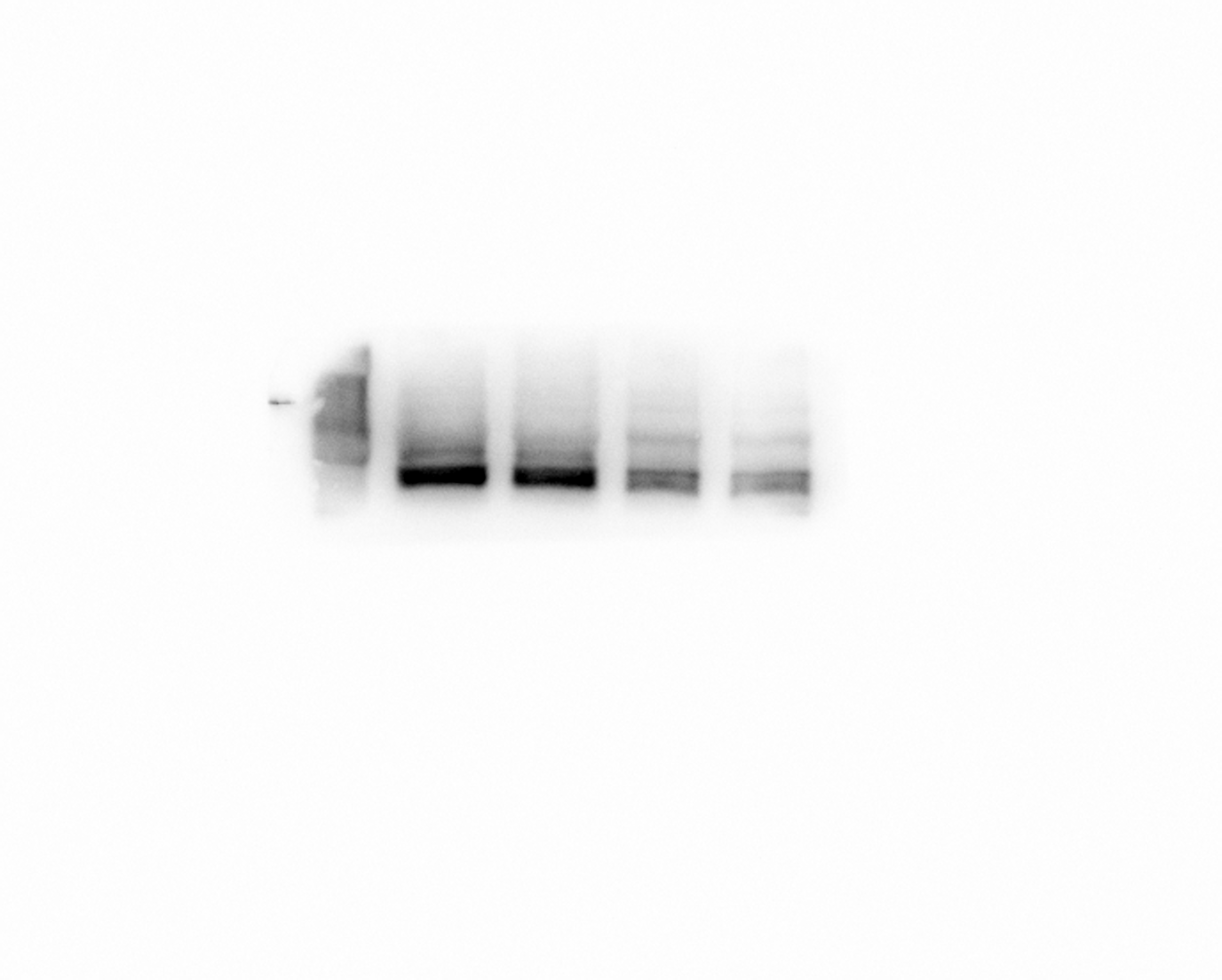

Supplement: Supplementary file 1 [file Data_Sheet_1.ZIP › Datasets/Western blot images/CHN1/CHN1(2W).Tif]

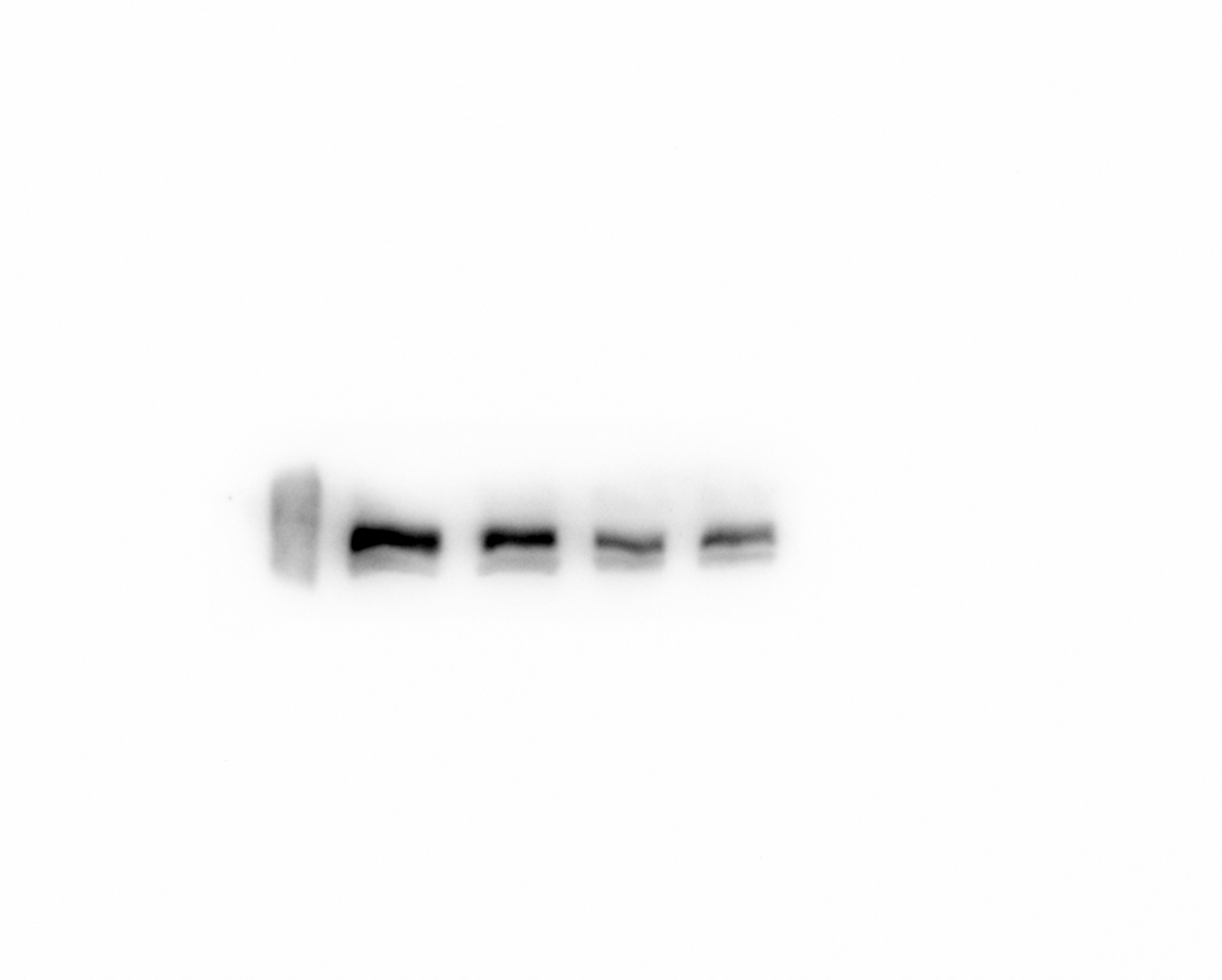

Supplement: Supplementary file 1 [file Data_Sheet_1.ZIP › Datasets/Western blot images/CHN1/CHN1(4W).Tif]

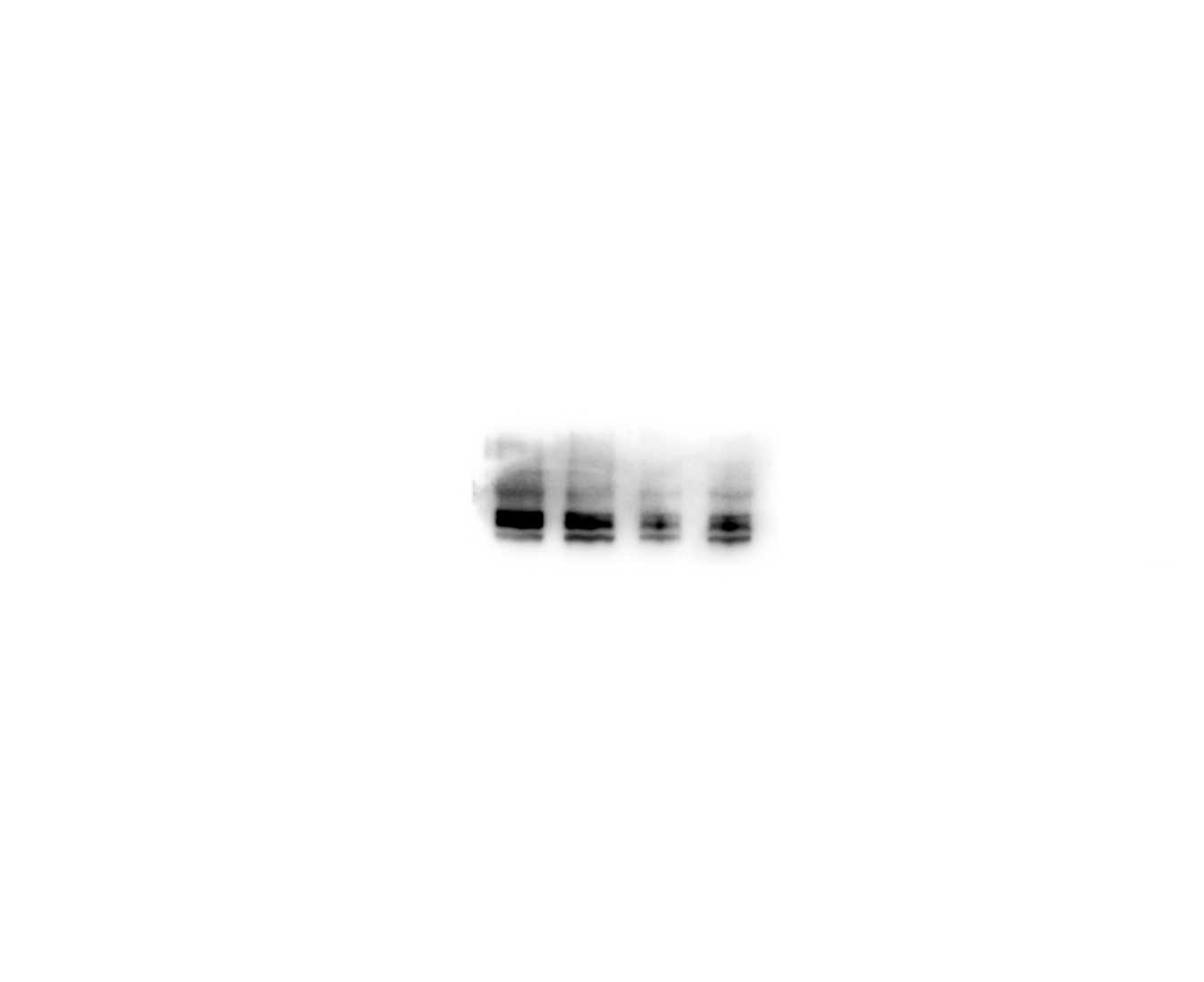

Supplement: Supplementary file 1 [file Data_Sheet_1.ZIP › Datasets/Western blot images/CHN1/CHN1(6W).Tif]

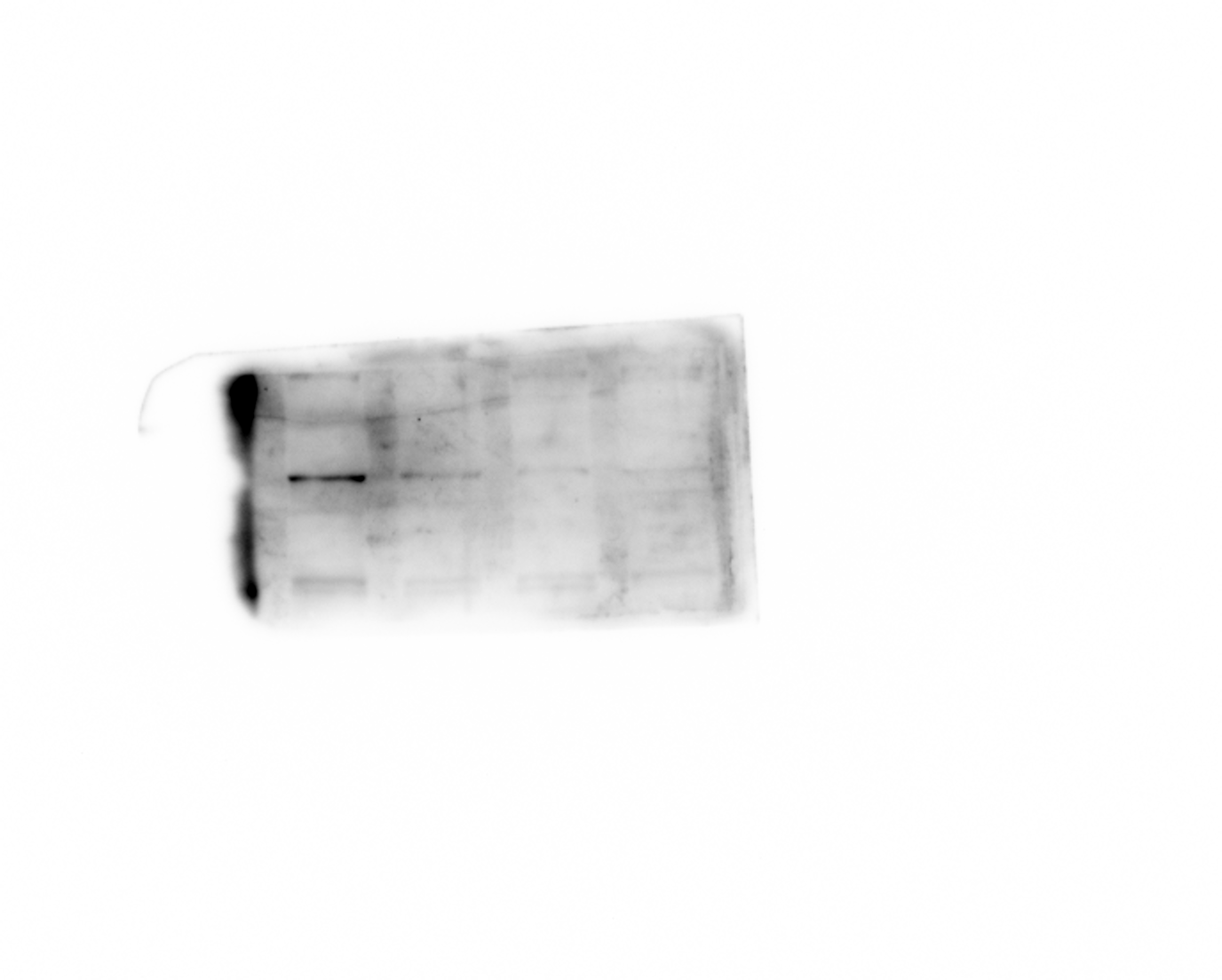

Supplement: Supplementary file 1 [file Data_Sheet_1.ZIP › Datasets/Western blot images/EphA4/2W.Tif]

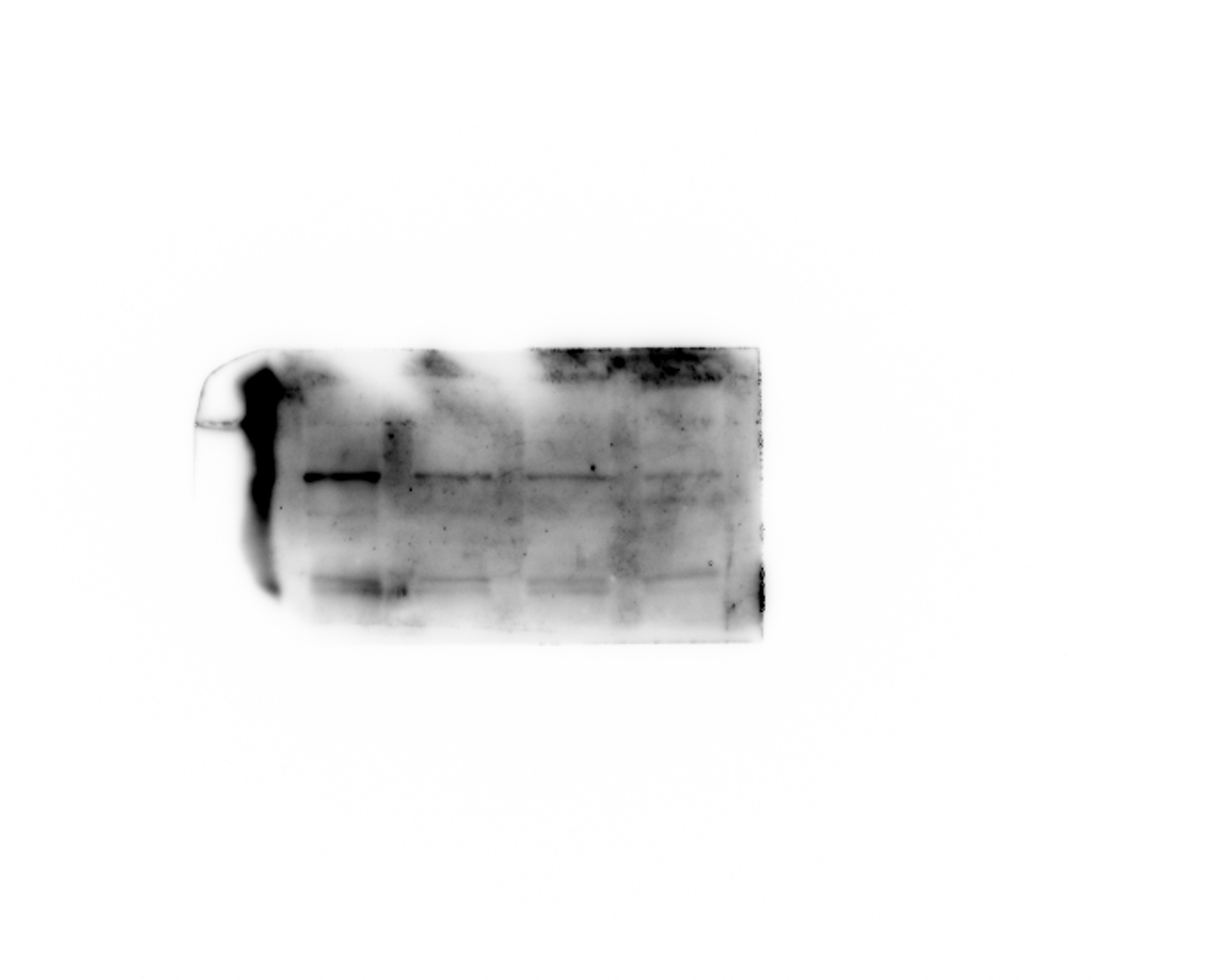

Supplement: Supplementary file 1 [file Data_Sheet_1.ZIP › Datasets/Western blot images/EphA4/4W.Tif]

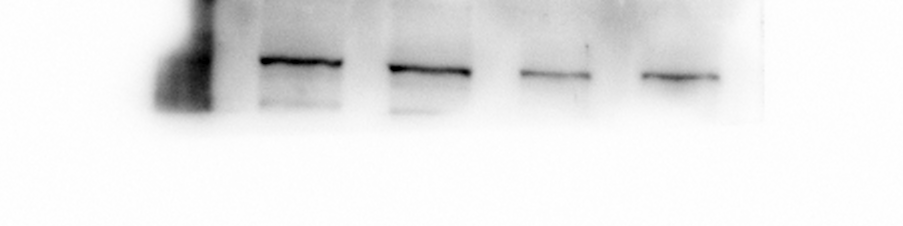

Supplement: Supplementary file 1 [file Data_Sheet_1.ZIP › Datasets/Western blot images/EphA4/6W.tif]

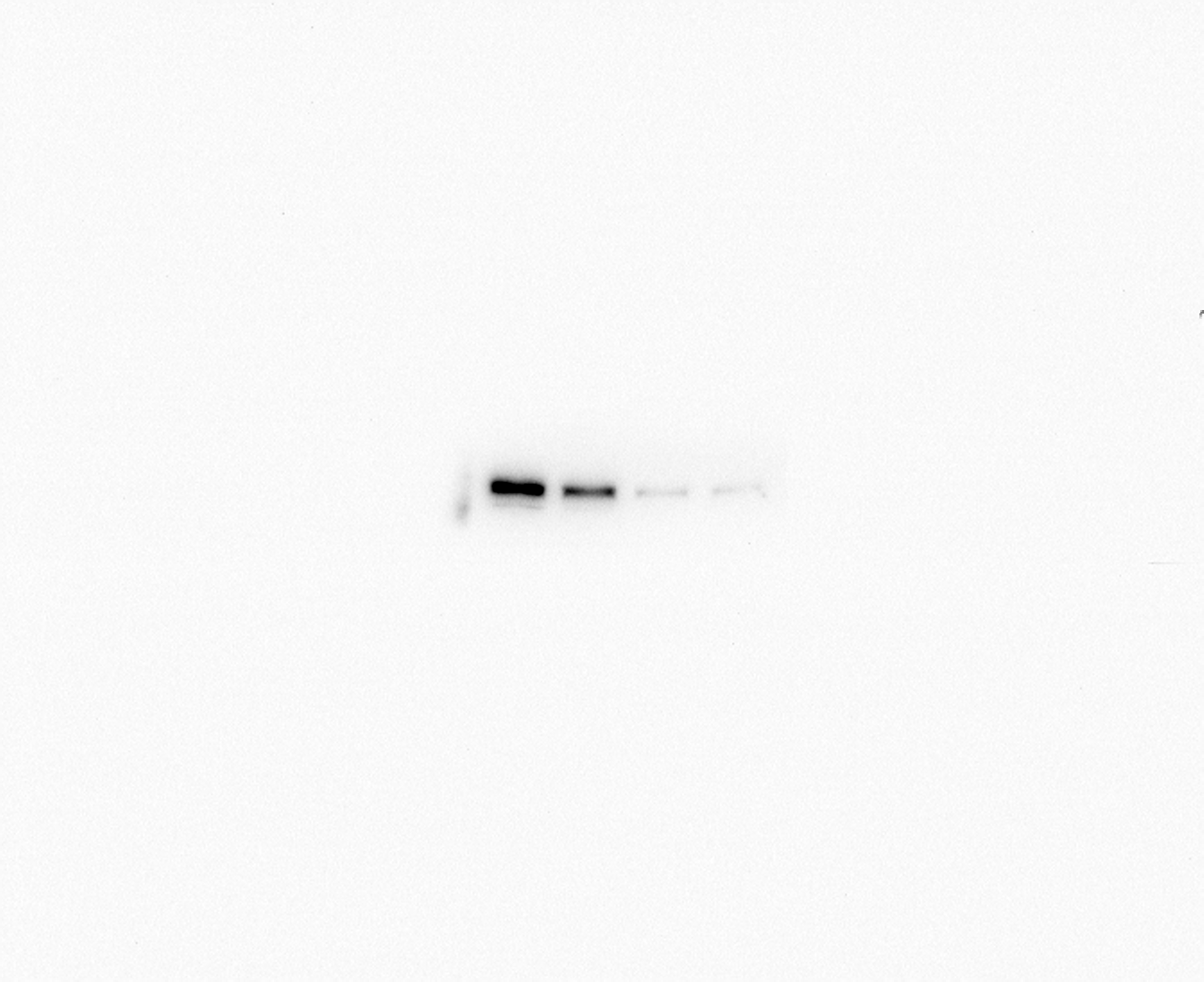

Supplement: Supplementary file 1 [file Data_Sheet_1.ZIP › Datasets/Western blot images/EphrinB3/2W.Tif]

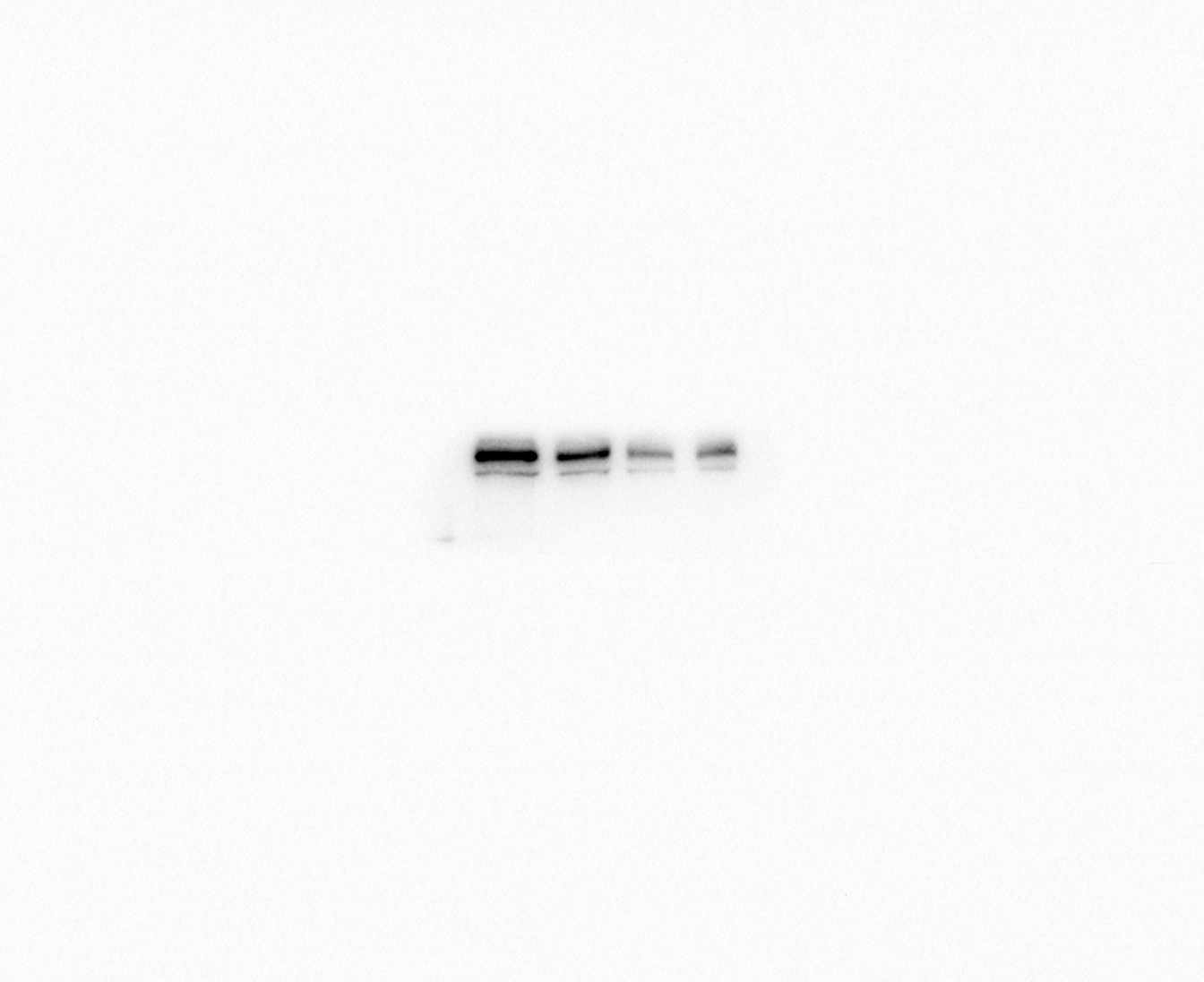

Supplement: Supplementary file 1 [file Data_Sheet_1.ZIP › Datasets/Western blot images/EphrinB3/4W.Tif]

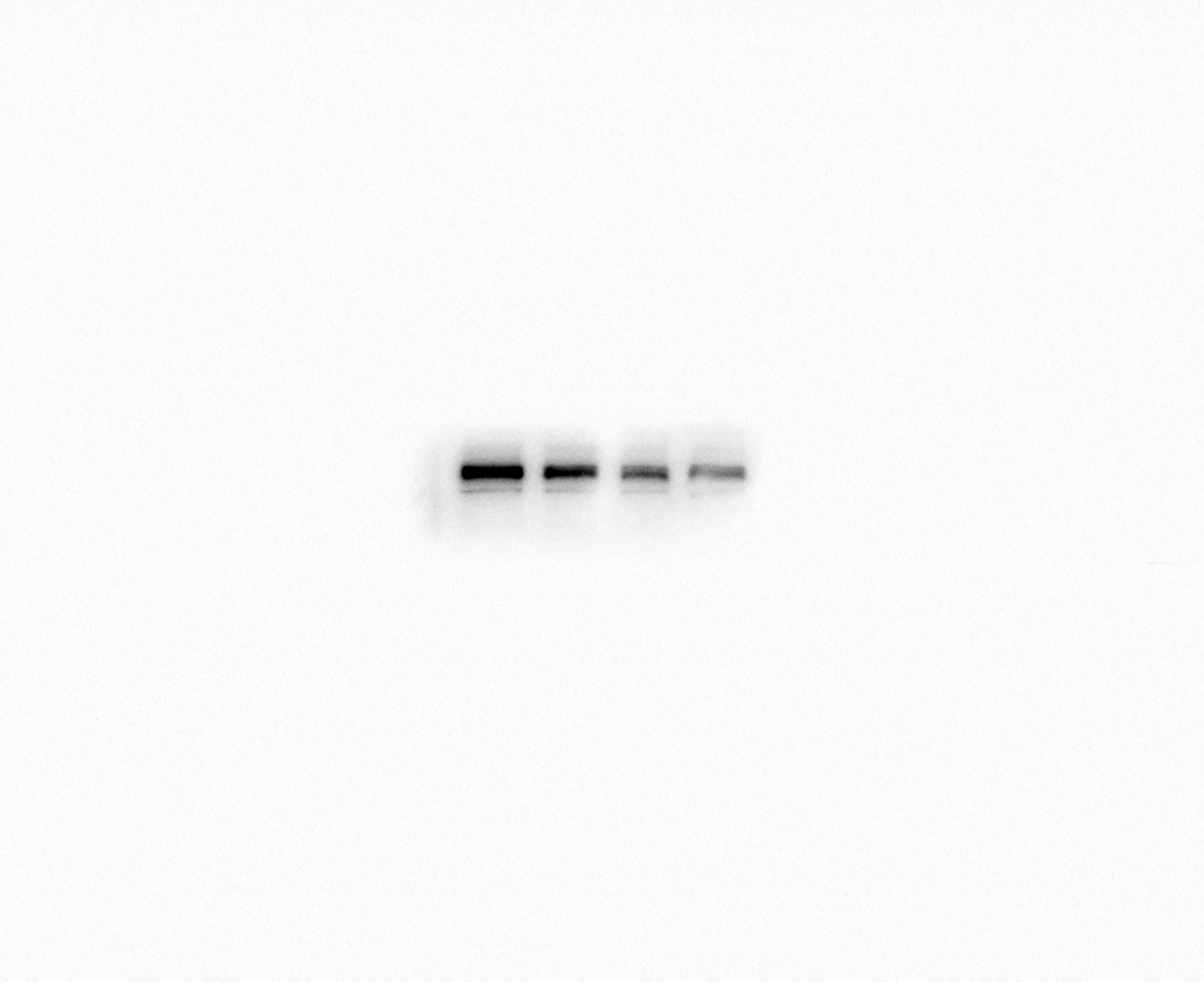

Supplement: Supplementary file 1 [file Data_Sheet_1.ZIP › Datasets/Western blot images/EphrinB3/6W.Tif]

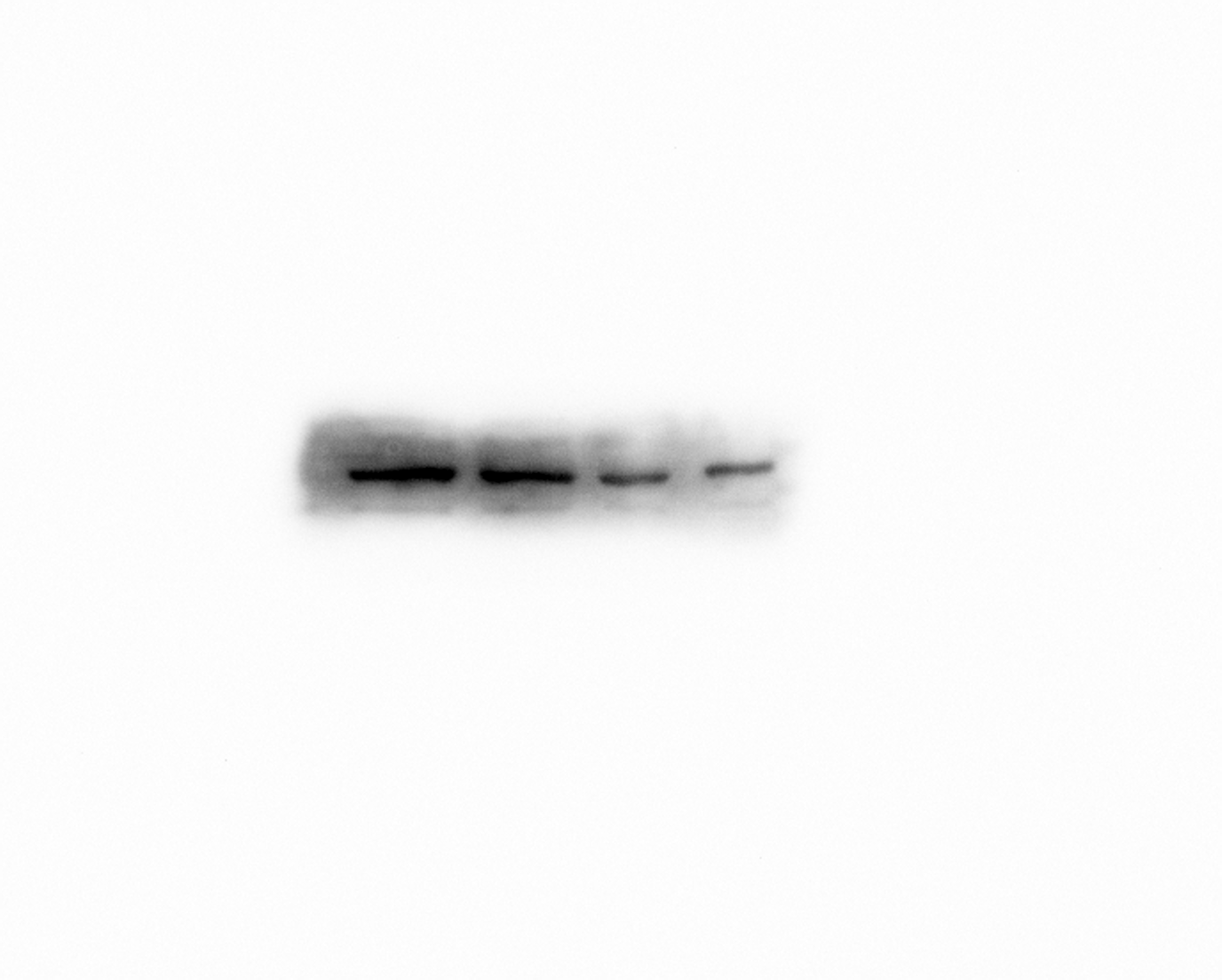

Supplement: Supplementary file 1 [file Data_Sheet_1.ZIP › Datasets/Western blot images/NCK1/NCK1(2W).Tif]

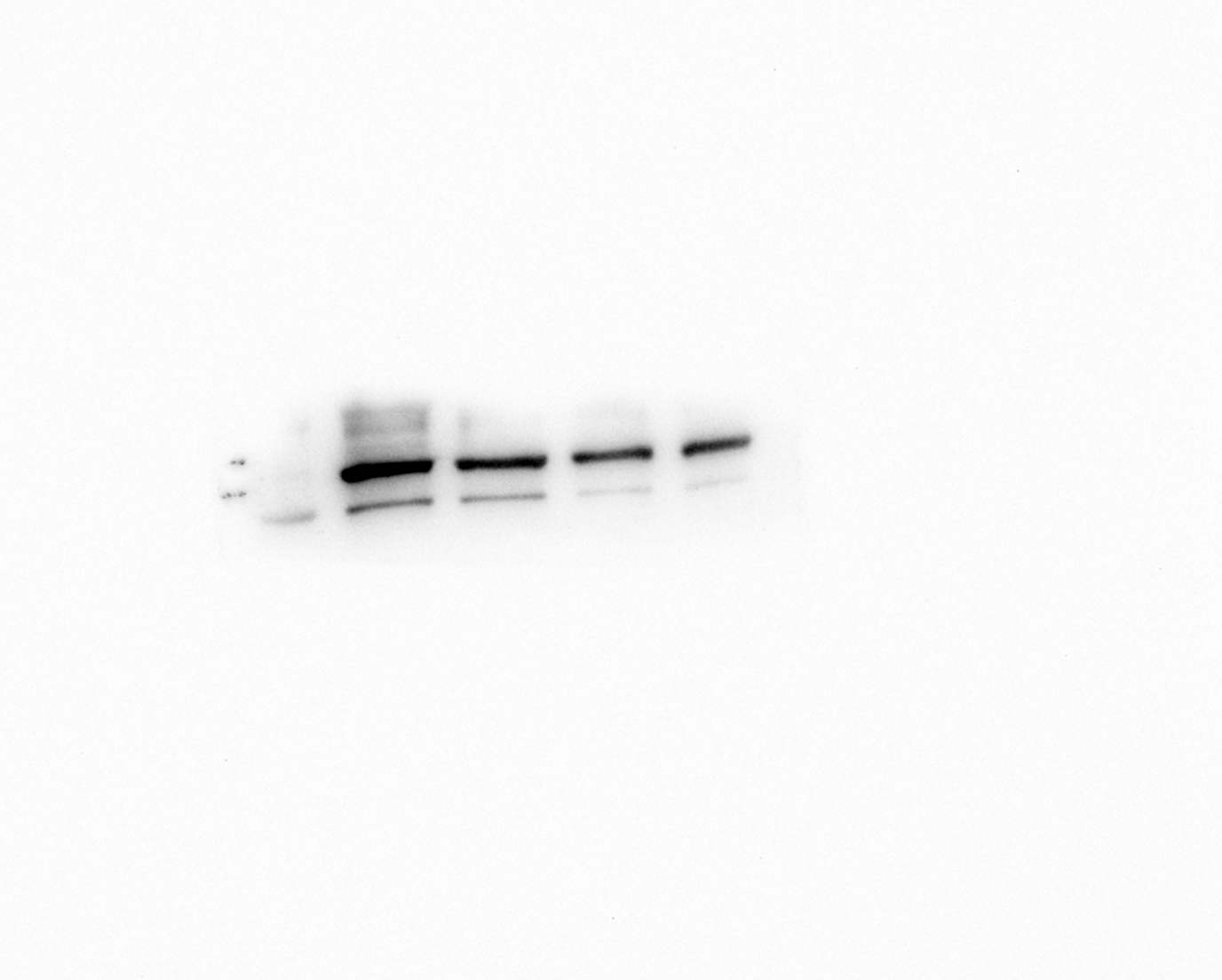

Supplement: Supplementary file 1 [file Data_Sheet_1.ZIP › Datasets/Western blot images/NCK1/Nck1(4W).Tif]

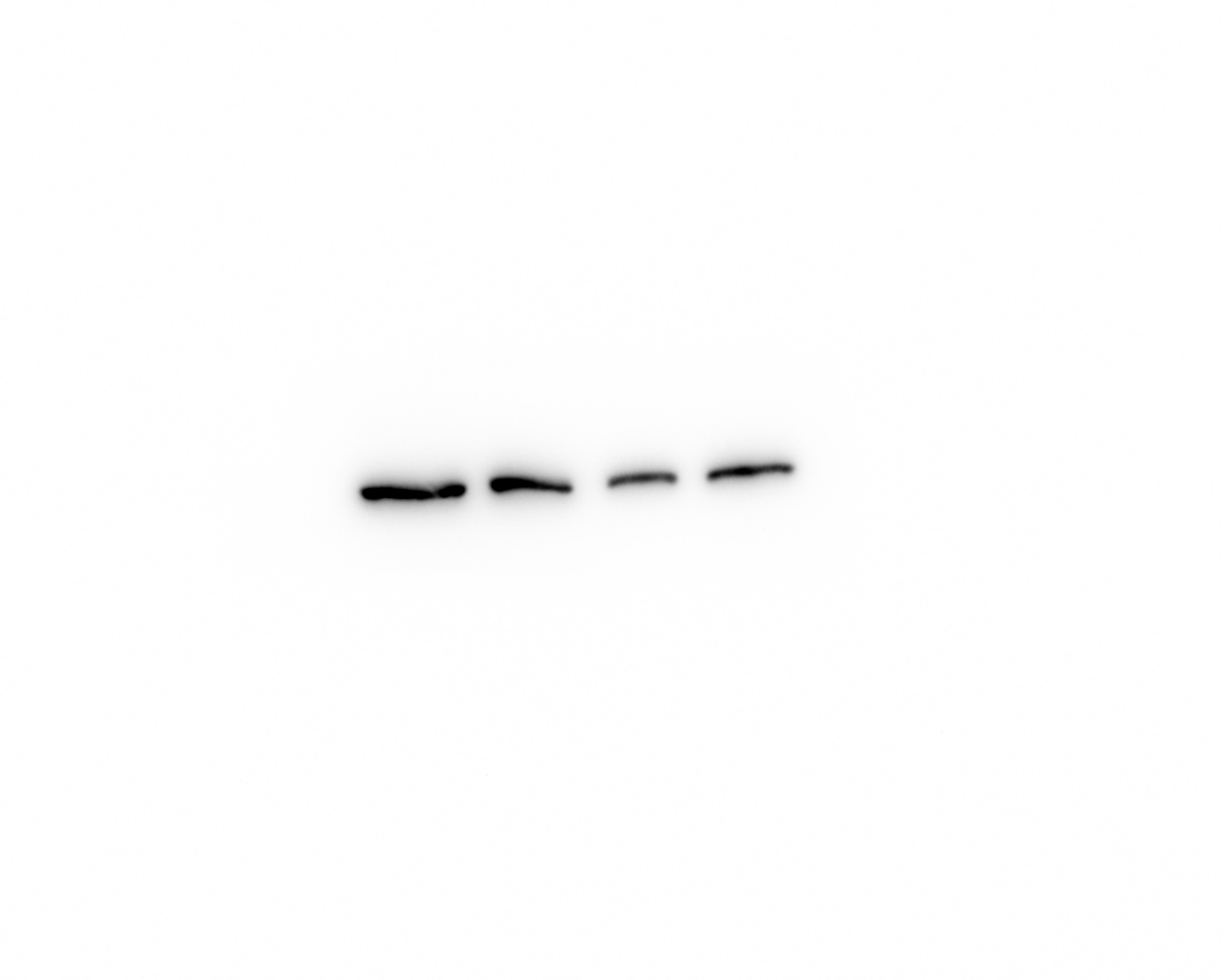

Supplement: Supplementary file 1 [file Data_Sheet_1.ZIP › Datasets/Western blot images/NCK1/NCK16Wú⌐.Tif]
